# Design and synthesis of betulinic acid–dithiocarbamate conjugates as potential antifungal agents against *Candida albicans*†

**DOI:** 10.1039/d4ra05020g

**Published:** 2024-12-03

**Authors:** Henna Amin, Hadiya Amin Kantroo, Mohamad Mosa Mubarak, Showkat Ahmad Bhat, Zahoor Ahmad, Khursheed Ahmad Bhat

**Affiliations:** a Bioorganic Chemistry Division, Indian Institute of Integrative Medicine (CSIR) Srinagar J&K 190005 India kabhat@iiim.res.in; b Clinical Microbiology and PK-PD Division, CSIR Indian Institute of Integrative Medicine Srinagar J&K 190005 India zap@iiim.res.in; c Academy of Scientific and Innovative Research (AcSIR) Ghaziabad-201002 India

## Abstract

Diverse betulinic acid–dithiocarbamate conjugates were designed and synthesized *via* a two-step reaction at room temperature. Among the fourteen dithiocarbamate analogs of betulinic acid, DTC2 demonstrated the best antifungal activity against *Candida albicans*, with a minimum inhibitory concentration (MIC) of 4 μg mL^−1^, achieving 99% fungicidal activity at the same concentration. These compounds were found to be ineffective against common Gram-negative and Gram-positive pathogens, suggesting their specificity to fungi. Furthermore, DTC2 exhibited synergistic effects with the antifungal drugs fluconazole and nystatin, resulting in a significant decrease in MIC by 64 and 16 folds, respectively, when co-administered. Notably, the molecule also hindered hyphae formation in *Candida albicans*, thereby reducing its pathogenicity. Furthermore, it displayed time- and concentration-dependent kill kinetics, sterilizing *C. albicans* within 8 hours at 8× MIC. Additionally, DTC2 exhibits greater efficacy against β-carbonic anhydrase with better docking scores and binding patterns than ethoxyzolamide, a well-known inhibitor of β-carbonic anhydrase.

## Abstract 2

Diverse betulinic acid–dithiocarbamate conjugates were designed and synthesized *via* a two-step reaction at room temperature.

## Graphical abstract

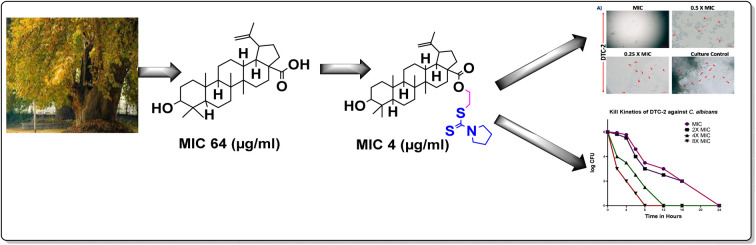

## Introduction

**Fig. 1:**
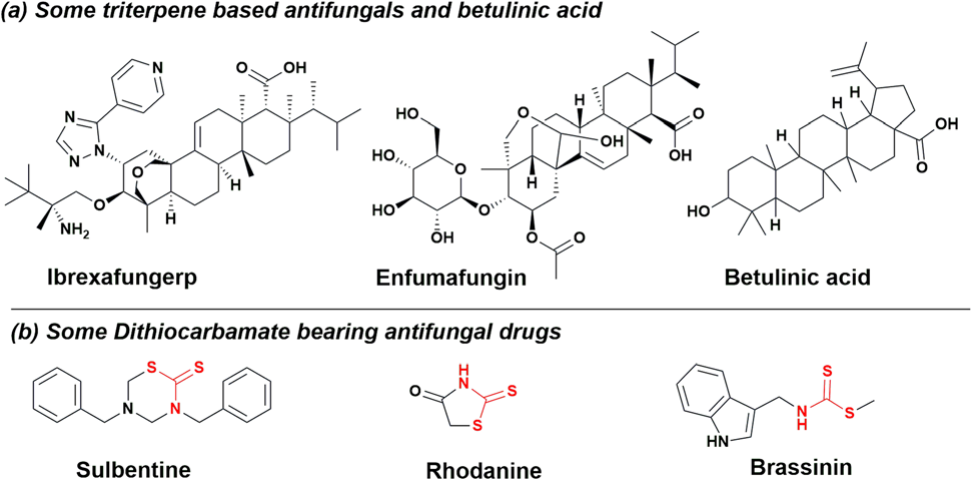
(a) Structures of betulinic acid, antifungal triterpenes and (b) dithiocarbamates antifungal compounds.

A recent increase in the prevalence of fungal infections has necessitated the need for the development of new antifungals. Fungal pathogens are responsible for approximately 13 million infections worldwide,^1^ accounting for an increasing mortality rate of around 1.5 million deaths annually.^2^ The number is expected to increase in the coming years due to the lack of potent therapeutics, shifts in the epidemiology of invasive fungal infections (IFI), a sharp surge in IFI among immunocompromised patients and the emergence of multidrug resistance.^3^ Candidiasis, a fungal infection, is caused by the overgrowth of *Candida*, predominantly *Candida albicans*, which is a leading causative agent responsible for approximately 70% of systemic candidiasis and mucosal infections. In normal individuals, it is usually found in association with microbial flora, while in the case of immunocompromised patients, it is an opportunistic pathogen. In the current scenario, three categories of drugs, *i.e.*, azoles (synthetic), polyenes and echinocandins (natural product), are used in the treatment of mycosis.^4^ The ability of *Candida albicans* to switch between different morphological forms, along with its resemblance to the human metabolic pathways, complicates the treatment of infections caused by this pathogen.^5^ This problem can be addressed by exploring novel antifungal agents and identifying the drug targets. Nature has consistently come to the aid of humans, as it harbours a vast array of chemically diverse molecules, known as natural products that exhibit potent biological activity. Griseofulvin, a polyketide, was the first natural product-based antifungal used systemically for the treatment of skin and nail infections.^6^ Pentacyclic triterpenes are the class of natural products that demonstrate a wide range of biological activity, such as anticancer,^7^ antiviral, antioxidant, and anti-inflammatory.^8^Fig. 1a depicts ibrexafungerp, a natural triterpene, presently undergoing phase III clinical trials for treating various fungal vulvovaginal conditions.^9^ Additionally, enfumafungin, another triterpenoid glycoside extracted from *Hormonema carpetanum*, exhibits potent antifungal properties.^10^ Betulinic acid (3-beta-hydroxy-lup-20(29)-en-28-oic acid), a pentacyclic lupane-type triterpenoid, is widely distributed throughout the plant kingdom. It is known for its anticancer,^11^ antiinflammatory,^12^ antidiabetic,^13^ antiHIV,^14^ antimalarial,^15^ and antidepressant properties.^16^ The modifications are usually carried out at C-3 hydroxyl, C-19 alkene and C-23 hydroxyl of betulinic acid to generate various functional derivatives.^17^ Dithiocarbamates are a group of sulphur containing compounds having predominant applications in the field of bioorganic and medicinal chemistry.^18^ The synthesis is usually carried out by reacting carbon disulphide and amine (primary or secondary) in the presence of electrophiles, such as imines, transition metals, epoxides, and alkyl halides. Dithiocarbamates possess potent antimicrobial properties due to the presence of an electron donating sulphur atom that forms chelates with positively charged metal, delocalizing electrons over the whole chelate ring. The sulphur atom reduces the polarity of the binding metal and thus increases the drug permeability into the microbial system.^19^ In certain cases, a hydrogen bond is usually formed between the binding site of the microbe and the –N–(S)SH group present in dithiocarbamates, causing an interruption in the normal physiology of the microbial system.^20^ It is an important scaffold in various antifungal drugs (Fig. 1b), such as sulbentine, rhodanine and brassinin.^21–23^

Considering the importance of triterpene and dithiocarbamate moieties as antifungals, we synthesized dithiocarbamate conjugates of betulinic acid to explore their antifungal activity against *Candida albicans*. Interestingly, among all the synthesized molecules, it was observed that DTC2 is highly potent against *Candida albicans*. Moreover, it showed synergism with the known antifungals (fluconazole and nystatin). A significant decrease in the MIC of these drugs was observed when taken in combination with DTC2. The docking studies further confirmed that DTC2 exhibited greater efficacy with a greater docking score and binding patterns against β-carbonic anhydrase than ethoxyzolamide.

## Results and discussion

Structural modification of bioactive natural products for enhancing pharmacological activities is an important research area in drug discovery.^24^ Herein, we design the structural modification of betulinic acid to fine tune its antifungal potential. Dithiocarbamates show diverse biological activities, such as anticancer, antifungal, antibacterial, antitubercular, antihyperglycemic, and antiinflammatory. Therefore, betulinic acid (BA) was modified to produce the dithiocarbamate conjugates. Betulinic acid was first esterified to obtain BE, followed by a reaction with CS_2_ and various secondary amines in the presence of K_2_HPO_4_ to obtain the desired derivatives. The reaction is facile with excellent yield. The products were identified using NMR and MS spectral data. The formation of intermediate compound BE is easily identified from the appearance of two new peaks at *δ* 63.3 and *δ* 27.5 in DEPT-NMR due to OCH_2_ and CH_2_Br, respectively. The molecular ion peak at *m*/*z* 562.30 further confirmed the formation of BE. The formation of the dithiocarbamate derivative DTC1 was ascertained from the diagnostic resonance peak at *δ* 194.4 due to the carbon–sulphur double bond. DTC1 was finally confirmed by its molecular ion peak at *m*/*z* 644.41 [M + H]^+^. Diverse dithiocarbamate derivatives of BA were prepared to study the effects of different substituents on antifungal activity. Different secondary amines, such as substituted piperidines, piperazines, pyrrolidines, thiomorpholine and morpholine were used to furnish the final compounds.

All the compounds were later screened for antimicrobial activity against *Candida albicans* (MTCC 183), *Escherichia coli* (MTCC 118) and *Staphylococcus aureus* (MTCC 737).

### *In vitro* activity of compounds against *C. albicans*

All the derivatives of betulinic acid (DTC1-14) were screened for antimicrobial activity against *C. Albicans* (Table 1). The results showed that most of the compounds possess moderate activity with an MIC of 64 or 128 μg mL^−1^. Only two compounds (DTC11 and DTC13) possess slightly better activity with an MIC of 32 μg mL^−1^. However, DTC2 displayed the best antifungal activity with an MIC value of 4 μg mL^−1^. The control drugs, fluconazole and nystatin, possess MIC values of 2 μg mL^−1^ and 4 μg mL^−1^, respectively.

**Table 1:** MIC values of the synthesized compounds measured in μg mL^−1^. MIC values of controls: fluconazole (0.039–20 μg mL^−1^) and nystatin (0.039–20 μg mL^−1^) were 2 μg mL^−1^ and 4 μg mL^−1^, respectively, against *C. albicans*

S. no.	Compound	MIC* (μg mL^−1^)	S. no.	Compound	MIC* (μg mL^−1^)
1	BA	64	9	DTC8	64
2	DTC1	128	10	DTC9	64
**3**	DTC2	**4**	11	DTC10	64
4	DTC3	128	12	DTC11	32
5	DTC4	64	13	DTC12	64
6	DTC5	64	14	DTC13	32
7	DTC6	64	15	DTC14	64
8	DTC7	64			

All the analogs except DTC2, DTC11 and DTC13 possess antifungal activity against *C. albicans* similar to that of parent molecule BA. Therefore, it can be inferred that the electronic structure of amine is essential for the enhanced activity of particular dithiocarbamate analogs. Furthermore, the cyclopropyl piperazine and bromo phenyl piperazine moieties of the carbamate group impart better activity to DTC11 and DTC13, respectively.

All the analogs except for DTC2 bear a six-membered heterocyclic ring, while DTC2 has five-membered pyrrolidine as part of the carbamate group. Thus, it clearly shows that pyrrolidine bearing carbamate is essential for activity.

### Activity against other pathogens (Gram-positive and Gram-negative bacteria)

After identifying the promising antifungal activity of DTC2 against *C. albicans*, we screened compounds (DTC, DTC1-14) against *E. coli* (Gram-negative) and *S. aureus* (Gram-positive) to explore their antibacterial potential. The results (Table 2) revealed that all the derivatives, including the parent compound, possess moderate activity against both Gram-positive and Gram-negative bacteria, with MIC values ranging from 64 to 128 μg mL^−1^.

**Table 2:** MIC values of the synthesized compounds measured in μg mL^−1^. MIC values of controls: Ciprofloxacin (0.039–20 μg mL^−1^) and Streptomycin (0.039–20 μg mL^−1^) were 1.25 μg mL^−1^ and 2.5 μg mL^−1^, respectively

S. no.	Compound	MIC^#^ (μg mL^−1^)		S. no.	Compound	MIC^#^ (μg mL^−1^)	
		*E. coli*	*S. aureus*			*E. coli*	*S. aureus*
1	BA	128	128	10	DTC9	128	128
2	DTC1	128	128	11	DTC10	64	128
3	DTC2	128	128	12	DTC11	128	128
4	DTC3	128	64	13	DTC12	128	128
5	DTC4	64	128	14	DTC13	128	128
6	DTC5	128	128	15	DTC14	128	128
7	DTC6	128	128	16	Ciprofloxacin	1.25	1.25
8	DTC7	128	64	17	Streptomycin	2.5	2.5
9	DTC8	128	128				

### Evaluation of full spectrum antifungal activity of this series of compounds

This series of compounds was evaluated against *Candida glabrata* and *Aspergillus* species, including *Aspergillus niger*, *Aspergillus flavus*, and *Aspergillus fumigatus*. The compounds exhibited moderate activities in the range of 64–128 μg mL^−1^ against these fungal strains.

### Activity against other pathogens (Gram-positive and Gram-negative bacteria)

After identifying the promising antifungal activity of DTC2 against *C. albicans*, we screened compounds (DTC, DTC1-14) against *E. coli* (Gram-negative) and *S. aureus* (Gram-positive) to explore their antibacterial potential. The results (Table 2) revealed that all the derivatives, including the parent compound, possess moderate activity against both Gram-positive and Gram-negative bacteria, with MIC values ranging from 64 to 128 μg mL^−1^.

### Minimum fungicidal concentration (MFC) of the lead compound

After confirming the promising fungistatic activity of DTC2, we delved into its fungicidal properties. Interestingly, the MFC value for DTC2 precisely matched its MIC, establishing an MIC to MFC ratio of 1 (<4). This indicates that DTC2 is fungicidal^25^ and can eradicate 99% of the pathogen at 4 μg mL^−1^. In comparison, controls fluconazole and nystatin exhibited an MFC to MIC ratio of 2 (Table 3), indicating their fungicidal nature.

**Table 3:** MFC values for DTC2 and controls fluconazole and nystatin against *C. albicans*

S. no.	Compound	MFC (μg mL^−1^)	MFC/MIC ratio
**1**	DTC2	**4**	**1**
2	Fluconazole	4	2
3	Nystatin	8	2

### Hyphal morphogenesis analysis

**Fig. 2:**
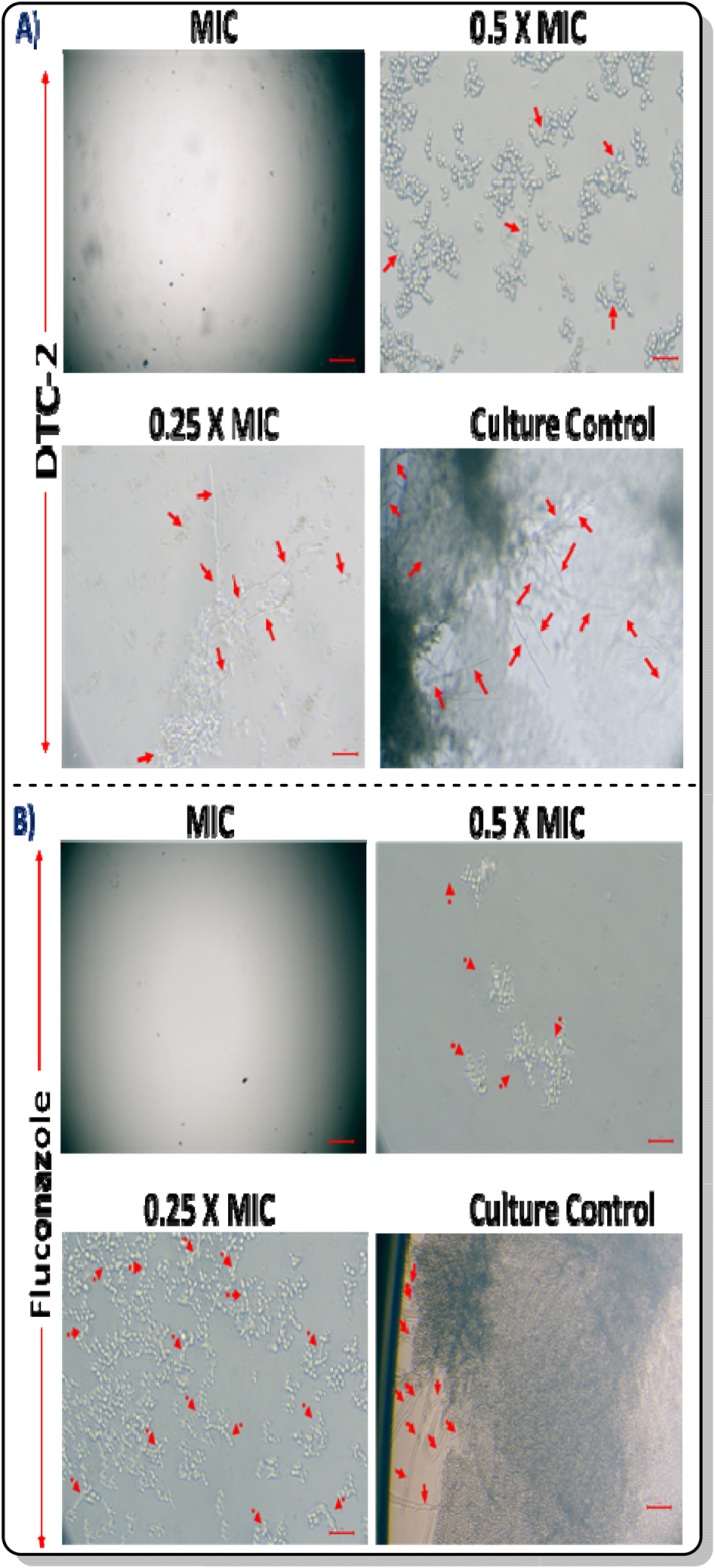
Hyphae inhibition in *C. albicans* by (A) DTC2 and (B) fluconazole.

As hyphae contribute to the pathogenicity of *C. albicans*, we investigated the impact of the lead compound on hyphal formation using fluorescence microscopy. Observations revealed diminished filamentation in *C. albicans* images with escalating concentrations of DTC2 akin to the fluconazole-treated group (Fig. 2). This suggests that the compound effectively mitigates the pathogenicity of *C. albicans*. Control wells displayed robust hyphal growth, while no culture was found in MIC wells. In wells at MIC and sub-MIC concentrations, inhibited hyphae formation is demonstrated with increasing drug concentration, which are indicated by red arrows.

### Evaluation of lead compounds with standard antifungal drugs

DTC2 was assessed in various combinations with established antifungals, fluconazole and nystatin, using the checkerboard microdilution assay. A notable decrease in the MIC of DTC2 was observed when combined with both fluconazole and nystatin. Specifically, in combination with fluconazole, the MIC of DTC2 decreased 64-fold, while the MIC of fluconazole decreased by 16-fold, resulting in a ∑FIC value of 0.0781 (<0.5), which indicates synergism (entry 1 in Table 4). Similarly, when combined with nystatin, the MIC of DTC2 decreased by 64-fold, and the MIC of nystatin decreased by 32-fold, yielding a ∑FIC value of 0.0468 (<0.5), which also indicates synergism (entry 2 in Table 4).

**Table 4:** Effect of DTC2 in combination with fluconazole (FLU) and nystatin (NYS) against *C. albicans*

S. no.	Drug/drug combination	MIC (μg mL^−1^)		FIC (μg mL^−1^)	∑FIC	Remarks
		Alone	Combination			
1	Fluconazole	2	0.125	0.0625	0.0781	Synergism
	DTC2	4	0.0625	0.0156		
2	Nystatin	4	0.125	0.03125	0.0468	Synergism
	DTC2	4	0.0625	0.0156		

### Time kill kinetics of DTC2 against *C. albicans*

**Fig. 3:**
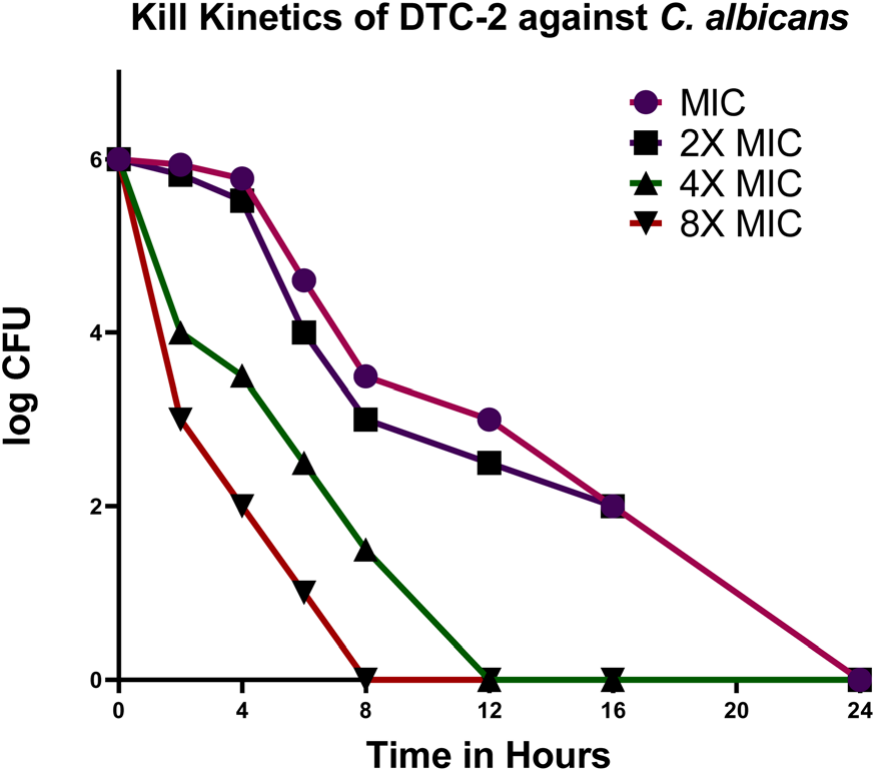
Impact of DTC2 on *C. albicans* examined over 24 hours with concentrations: MIC (magenta), 2× MIC (violet), 4× MIC (green), and 8× MIC (maroon).

We conducted kill kinetics studies on DTC2, revealing intriguing results. At both the MIC and 2× MIC, the drug exhibited a 2-log reduction in microbial count by the 8th hour and a 4-log reduction by the 16th hour, effectively sterilizing the pathogen by the 24th hour. Conversely, at 4× MIC, complete sterilization was achieved by the 12th hour, while at 8× MIC, it occurred as early as the 8th hour (Fig. 3). These findings underscore DTC2's time and concentration-dependent bactericidal activity against *C. albicans*, characterized by a progressive decrease in log values with increasing time and concentration. Understanding time-kill kinetics is crucial because it provides insights into how the antimicrobial impact on microbial elimination or growth inhibition changes over time. Such knowledge is essential for optimizing dosage regimens in therapeutic drug monitoring, aiding in determining the optimal timing and frequency of drug administration to maintain therapeutic levels throughout the dosing interval.^26^

### Cytotoxicity assay

**Fig. 4:**
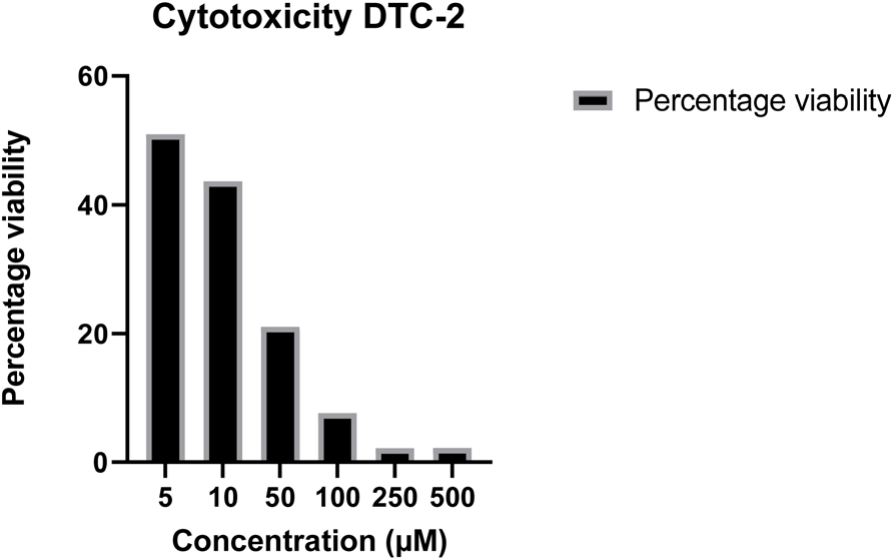
Cell viability percentage of HEK-298 cell lines on exposure to DTC2 at concentrations 5 μM, 10 μM, 50 μM, 100 μM, 250 μM, and 500 μM.

The cytotoxicity of the most potent antifungal molecule, *i.e.*, DTC2, was evaluated on HEK-293 cells using an MTT assay at concentrations ranging from 5 μM to 500 μM, and cell viability was measured after 24 hours of incubation. At higher concentrations, a notable reduction in cell viability was observed; at 50 μM, cell viability decreased by 32.26%. In contrast, at lower concentrations, especially those matching MIC concentrations, magnificent cell viabilities of 64.3% for 10 μM and 73.69% for 5 μM (Fig. 4) were observed. These results indicate that DTC2 exhibits significantly lower toxicity near its antifungal concentrations, maintaining over 70% cell viability.

### Molecular docking and mechanistic potential of DTC2

**Fig. 5:**
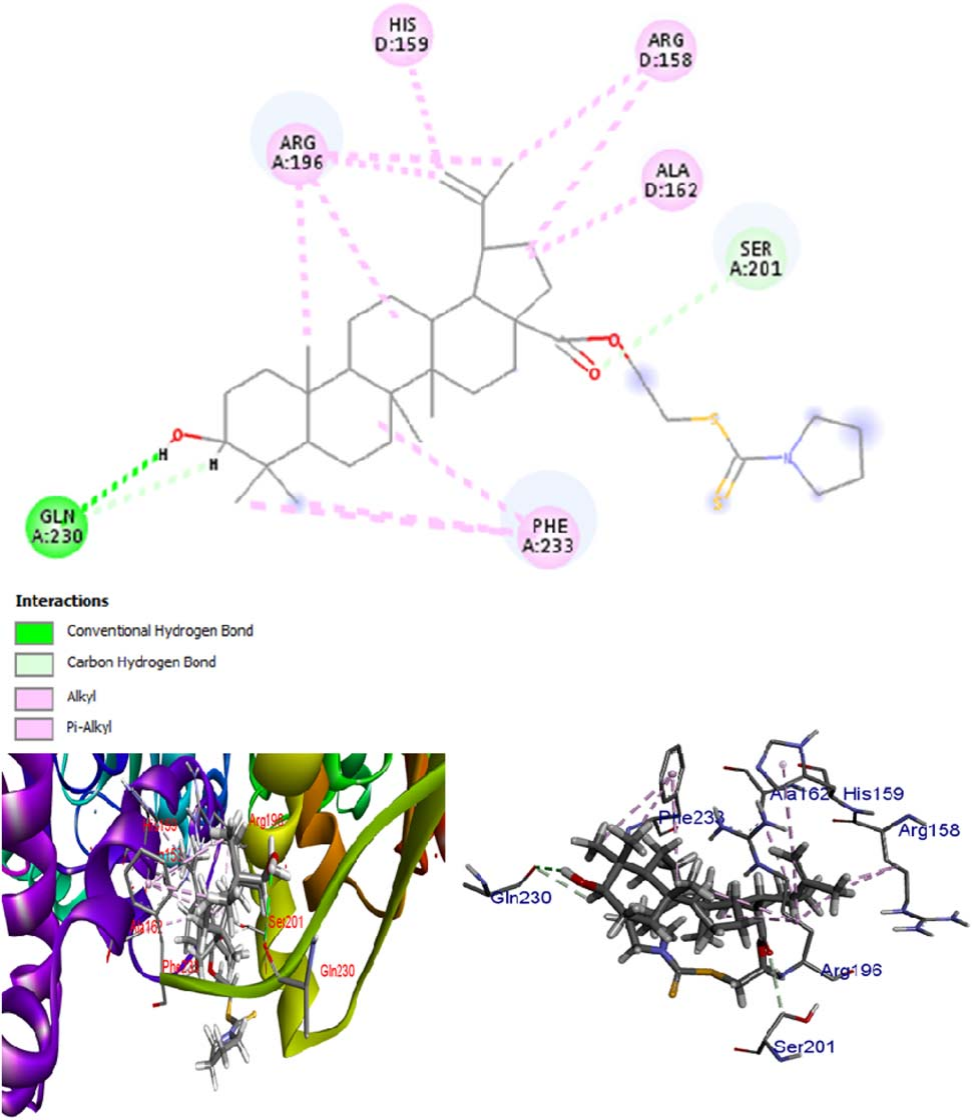
Binding of DTC2 to β-carbonic anhydrase of *C. albicans*, a key enzyme that helps in the survival of this pathogen under different CO_2_ concentrations.

A docking study was performed for lead molecule DTC2 against β-carbonic anhydrase of *C. albicans*, an enzyme crucial for the pathogen's survival under varying CO_2_ concentrations. The results showed that DTC2 exhibited a strong binding affinity for β-carbonic anhydrase, with a docking score of −384.47 kJ mol^−1^. It is worth mentioning that DTC2 significantly outperformed a well-known inhibitor of carbonic anhydrase, ethoxyzolamide, with only a docking score of −216.62 kJ mol^−1^. The compound interacts with several residues, such as glutamine (A:230), phenylalanine (A:233), and arginine (A:196), with an extensive network of pi–alkyl and hydrogen bonds. The hydrogen bond with glutamine (A:230) and additional pi–alkyl interactions with arginine and histidine indicate a more hydrophobic binding pocket. The control, ethoxyzolamide, shows hydrogen bonding (conventional and carbon–hydrogen bonds) with asparagine (A:151, C:151), glycine (C:147, A:148), and lysine (A:145) while exhibiting pi–anion interactions with asparagine (A:151) and pi–alkyl interactions with leucine (A:152) and valine (C:149). This indicates a binding pocket with both hydrophilic and hydrophobic interactions. Overall, the DTC2 shows a more robust network of hydrophobic interactions compared to ethoxyzolamide, suggesting that it may have a more significant binding affinity for hydrophobic regions of the substrate (Fig. 5). Our study aligns with previous studies, showing that betulinic acid is ineffective against *C. albicans*. However, modifications at the 28-C position of the compound demonstrated improved activity against *C. albicans*.^27^ Similarly, in our study, the addition of a dithiocarbamate group at the C-28 position enhanced the activity, likely by helping the compound reach the target.

## Experimental section

### General information

All the necessary reagents and solvents were purchased from Sigma-Aldrich and TCI. UV cabinet (camag) was used to visualise spots on TLC. All the products were purified using silica gel (60–120) column chromatography. ^1^H NMR and ^13^C NMR spectra were recorded in CDCl_3_ using 400 and 125 MHZ NMR spectrometers, respectively. Chemical shifts of ^1^H and ^13^C NMR were expressed in parts per million (ppm).

### Experimental procedures

#### Procedure for isolation of betulinic acid from *Platanus orientalis*

**Fig. 6:**
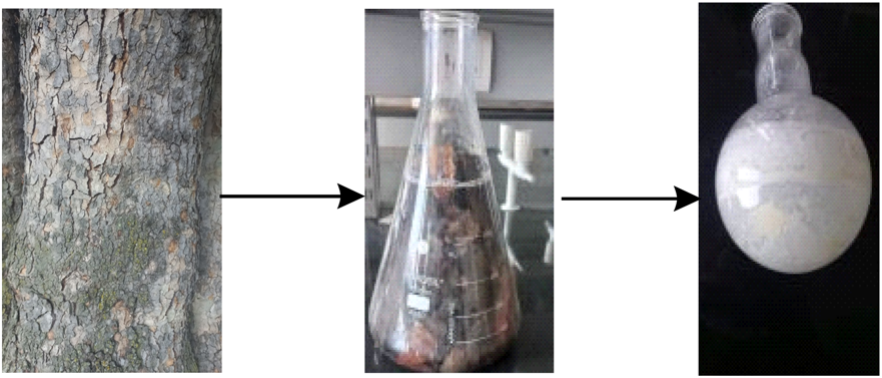
Procedure for the isolation of betulinic acid.

The dry bark of *Platanus orientalis* was taken and chopped into small pieces and meshed to powder form. The powdered plant material was then put into a 2.5 L conical flask containing chloroform and was kept for 24 hours. The mother liquor was then filtered through Whatman filter paper and consequently concentrated under reduced pressure. The concentrate was further washed with hexane to remove some nonpolar impurities. The extract thus obtained was purified using silica gel column chromatography, and pure betulinic acid molecules (BA) (12 grams) were isolated with increasing polarity of hexane:ethyl acetate solvent system (Fig. 6).

#### Procedure for the synthesis of BE

To a 50 mL round bottom flask containing 20 mL DMF, betulinic acid (1 g, 2.1 mmol) and potassium carbonate (290.2 mg, 2.1 mmol) were added to 1,2-dibromoethane (0.78 g, 4.2 mmol). The mixture was stirred at 40 °C for 6–8 hours. The progress of the reaction was monitored using TLC. After the completion of the reaction, ice cold water was added to the reaction mixture and extracted with EtOAc. The process was repeated thrice, and the organic layers were pooled and concentrated under a vacuum to yield the residue. The residue thus obtained was purified using silica gel column chromatography (mesh size 60–120) and hexane–EtOAc as an eluent to obtain the desired product (BE) with 80% yield (0.94 grams).

#### Synthesis of dithiocarbamate conjugates of betulinic acid

**Scheme 1:**
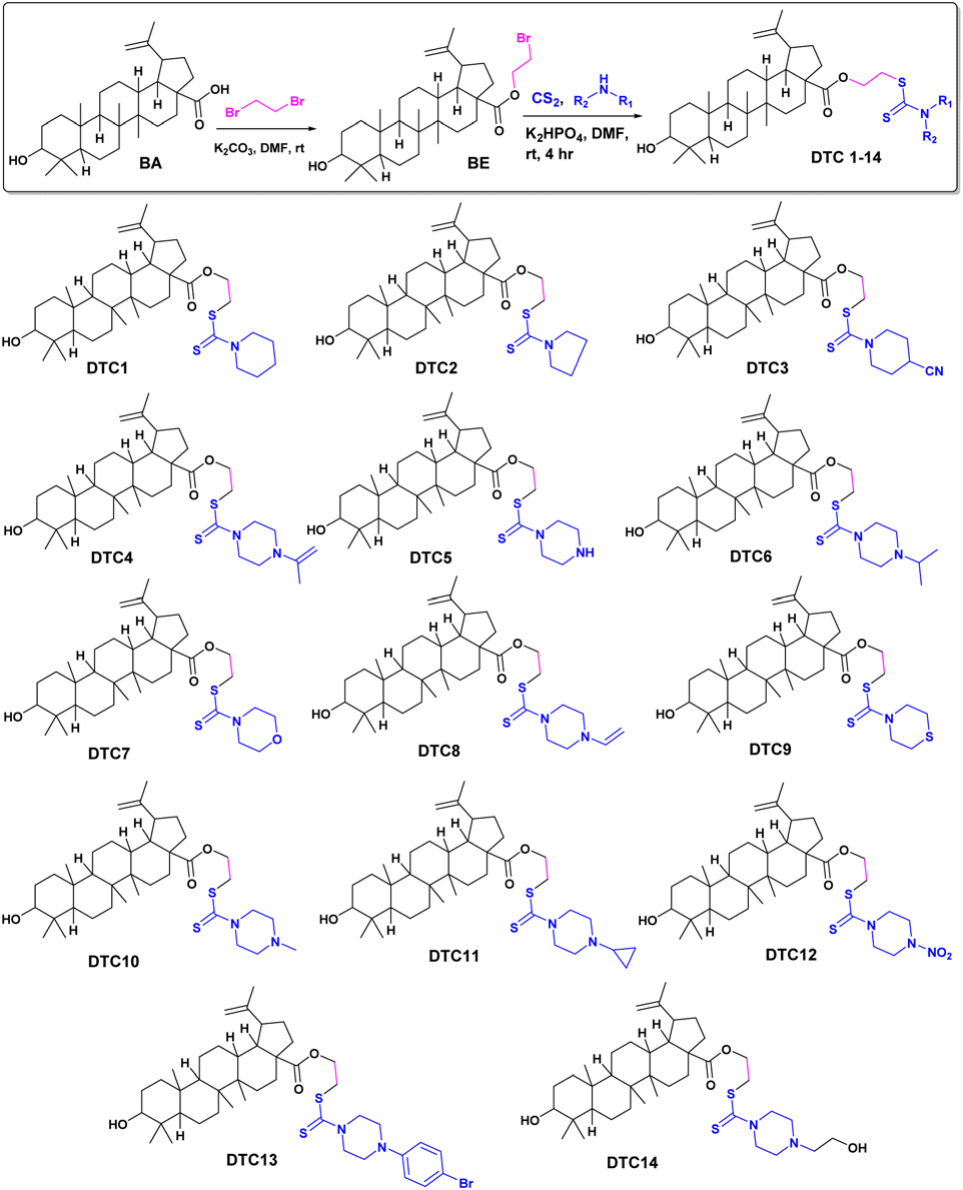
Synthesis of various dithiocarbamate conjugates of betulinic acid.

A solution of secondary amine (0.12 mmol, 1.2 eq.) (Scheme 1) in DMF (3 mL) was added CS_2_ (0.12 mmol, 1.2 eq.) and K_2_HPO_4_ (0.15 mmol, 1.5 eq.) in a 25 mL round bottom flask. The reaction mixture was stirred at room temperature for 30 minutes, followed by the addition of BE (0.1 mmol, 1.0 eq.). The reaction mixture was further stirred for 4–5 hours. The progress of the reaction was monitored using TLC. After completion of the reaction, ice cold water was added to the reaction mixture and extracted with EtOAc. The organic layer was dried using sodium sulphate, concentrated over reduced pressure and purified through silica gel column chromatography (mesh size 60–120) and hexane–EtOAc as eluent to obtain the desired products, DTC1–DTC14.

#### Chemicals and reagents for the determination of antifungal and antibacterial activity of betulinic acid analogs (DTC1–DTC14)

Sabouraud Dextrose Agar (SDA), Sabouraud Dextrose Broth (SDB), Muller Hinton Broth (MHB), and Nutrient Agar (NA) were procured from HiMedia, while RPMI-1640 was obtained from Sigma-Aldrich, and 96 well plates were from Tarsons. All the test samples, parent compounds as well as reference antifungal agents (fluconazole (FLU) and nystatin (NYS)) and antibacterial agents (Ciprofloxacin (CIP) and Streptomycin (STR)) were prepared as 10 mg mL^−1^ stock solutions in dimethyl sulfoxide (DMSO) and stored at 4 °C.

#### Fungal and bacterial strains

The fungal strain *Candida albicans* (MTCC 183), *Candida glabrata* (MTCC 3019), *Aspergillus niger* (MTCC 281), *Aspergillus fumigatus* (MTCC 8248), *Aspergillus flavus* (MTCC 3783), bacterial strains *Escherichia coli* (MTCC 118) and *Staphylococcus aureus* (MTCC 737) were obtained from CSIR-IMTech and maintained at −80 °C. For each experiment, the strains were thawed and cultured in the respective broths (SDB for the fungal strain and MHB for the bacterial strains) until they reached an optical density (OD 600) of 0.4 to 0.5.

#### Determination of minimum inhibitory concentration (MIC)

MIC was determined using the broth micro-dilution method, following established procedures for fungi^28^ and bacteria,^29^ respectively. In brief, for fungi, RPMI medium with serial two-fold concentrations ranging from 0.25 to 128.00 μg mL^−1^ of compounds DTC1–DTC14 was used. Approximately 1 × 10^3^ cells of *Candida* species and 1 × 10^4^ cells of *Aspergillus* species were inoculated in individual wells of a 96-well plate. Control wells contained fluconazole, nystatin, and Itraconazole at concentrations ranging from 0.078 to 20 μg mL^−1^. For bacteria, MHB containing serial two-fold concentrations from 0.25 to 128.00 μg mL^−1^ of DTC1 to DTC14 were separately inoculated with ∼1 × 10^5^ cells of *Escherichia coli* and *Staphylococcus aureus* in a 96-well plate. Control wells contained Ciprofloxacin (CIP) and Streptomycin (STR) at concentrations ranging from 0.078 to 20 μg mL^−1^. After 24 hours of incubation at 37 °C, the plates were inspected for visible growth.

#### Determination of minimum fungicidal concentration (MFC) for lead compound

MFC was assessed following MIC determination, as done previously.^30^ Wells, showing no growth at MIC and above MIC (2×, 4×, 8×, and 16× MIC) of DTC2, fluconazole and nystatin, were plated onto Sabouraud dextrose agar. Incubation at 37 °C for 24 hours preceded colony counting to determine MFC, which is crucial for evaluating antifungal efficacy. MFC was the concentration at which 99% killing of pathogens was observed. The MIC/MFC ratio was computed to discern the fungistatic or fungicidal nature of DTC2.

#### *C. albicans* morphogenesis analysis

*C. albicans* cells were exposed to DTC2 at MIC and sub-MIC concentrations (MIC/2, MIC/4) and incubated for 24 hours at 37 °C in a 12-well plate. Following this incubation, a detailed microscopic examination of the plate was conducted to evaluate the impact of DTC2 treatment specifically on hyphae formation within the *Candida albicans* cells (the imaging system model used was CH30RF200 from Olympus Optical Co. Ltd, Shinjuku, Tokyo, Japan. It was coupled with a Magnus camera (Magcam-DC, Central Honsho, Japan). The images showcased were taken at a magnification of 40×). This approach aimed to elucidate the antifungal effects of DTC2 on the morphological characteristics and growth patterns of the treated *C. albicans* cells.^31^

#### Drug–drug interaction with known antibiotics

To elucidate the interaction dynamics and efficacy of DTC2 with established antifungal agents (fluconazole and nystatin), a Checkerboard Micro Dilution Assay was conducted, as done previously.^32^ The assay spanned a range covering their MICs. In 96-well plates, DTC2 underwent horizontal serial dilution, while the established antifungal agent fluconazole/nystatin underwent vertical dilution, creating a checkerboard configuration of the combined concentrations. Each well received an inoculum of *C. albicans*, with a log 10 CFU value of 103. Following a 24 hour incubation at 37 °C, the Fractional Inhibitory Concentration Index (FICI) was determined (FICI = FICIA + FICIB). The ∑FIC, obtained by adding the FIC of DTC2 to that of FLU/NYS, was utilized to assess synergy (≤0.5), additivity or no interaction (0.5–4), or antagonism (≥4) in the combined concentrations of DTC2 and fluconazole/nystatin.

#### Time kill kinetics

To investigate the fungicidal mechanism of DTC2, a time-kill kinetics assay was conducted following CLSI guidelines.^33^ Tubes containing SDB with varying DTC2 concentrations (1× MIC, 2× MIC, 4× MIC, and 8× MIC) were inoculated with *C. albicans* at a density of 10^6^ cells per mL. Incubation at 37 °C for 24 hours was followed by viability checks at 0, 2, 4, 8,12, 16, and 24 hours. Serial dilutions in normal saline solution (NSS) were plated on SDA, and after subsequent 24 hour incubation at 37 °C, colony counts were determined.^34^ Log 10 reductions were calculated for each time interval, and a CFU *versus* time graph was generated using GraphPad Prism 9.0. Three independent experiments were performed, and the mean data were plotted.

#### Cytotoxicity assay

The cytotoxicity of the lead compound, DTC-2, was evaluated using the MTT assay on HEK-293 cells (human embryonic kidney cells). Cells in their logarithmic growth phase were harvested, counted, and seeded at a density of 10^4^ cells per well in 100 μL of culture medium in a 96-well microtiter plate. After overnight incubation at 37 °C with 5% CO_2_ to allow cell attachment, the cells were exposed to different concentrations of DTC-2 (5, 10, 50, 100, 250, and 500 μM) in triplicate. The treated cells were incubated for an additional 24 hours under the same conditions. MTT solution (20 μL) was then added to each well, and the plates were shaken at 150 rpm for 5 minutes to ensure thorough mixing. After 1–5 hours of incubation at 37 °C with 5% CO_2_ to allow the MTT to be metabolized, the media was removed, and 200 μL of DMSO was added to dissolve the formazan crystals. Absorbance at 560 nm was measured using an ELISA plate reader, and cell viability percentages were calculated.^35^

#### Molecular docking

The crystal structure of β-carbonic anhydrase was retrieved from the RCSB PDB database, while the structure of ethoxyzolamide was obtained from PubChem. 3D models of DTC-2 and fluconazole were generated using ChemBioDraw3D Pro and subsequently energy-minimized in PDB format. Molecular docking of β-carbonic anhydrase with the ligands DTC-2 and ethoxyzolamide was conducted separately using Hex 8.0, with a 0.6 Å grid applied across all ligand–receptor coordinates. Docking parameters, including translational steps, protein flips, and twist range, were set to 360°. The resulting docked models were analyzed using Discovery Studio Visualizer to examine the 3D structures and visualize the binding interactions.^36^

### Analytical data

#### BE: 2-Bromoethyl 9-hydfroxy-5*a*,5*b*,8,8,11*a*-pentamethyl-1 (prop-1-en-2-yl)icosahydro-3*aH*-cyclopenta[*a*]chrysene-3*a*-carboxylate

The product was purified by column chromatography using hexane : EtOAc (94 : 6) to afford BE as a pale white solid, displayed molecular ion peak at *m*/*z* 562.30 [M]^+^ in ESIMS, corresponding to molecular formula C_32_H_51_BrO_3_. ^1^H NMR (400 MHz, CDCl_3_) *δ* 4.67 (s, 1H), 4.60–4.50 (m, 1H), 4.33 (dd, *J* = 10.9, 5.8 Hz, 2H), 3.47 (t, *J* = 5.9 Hz, 2H), 3.12 (dd, *J* = 11.2, 5.0 Hz, 1H), 3.07–2.85 (m, 1H), 2.25–2.19 (m, 1H), 2.12 (td, *J* = 12.7, 3.6 Hz, 1H), 1.89–1.80 (m, 2H), 1.72 (s, 1H), 1.65 (d, *J* = 2.4 Hz, 1H), 1.62 (s, 3H), 1.58 (d, *J* = 5.2 Hz, 1H), 1.53 (dd, *J* = 7.6, 3.9 Hz, 2H), 1.48–1.40 (m, 2H), 1.40–1.32 (m, 5H), 1.30 (s, 2H), 1.18 (s, 3H), 1.12–1.07 (m, 1H), 0.96 (d, *J* = 12.4 Hz, 1H), 0.90 (d, *J* = 2.6 Hz, 6H), 0.85 (s, 3H), 0.82 (d, *J* = 4.6 Hz, 1H), 0.75 (s, 3H), 0.68 (s, 3H), 0.61 (d, *J* = 9.0 Hz, 1H); ^13^C NMR (101 MHz, CDCl_3_) *δ* 175.76, 150.46, 109.70, 79.02, 63.35, 56.69, 55.34, 50.55, 49.42, 46.96, 42.41, 40.74, 38.86, 38.79, 38.34, 37.19, 37.01, 34.31, 32.07, 30.59, 29.68, 29.19, 27.99, 27.38, 25.53, 20.89, 19.38, 18.29, 16.15, 16.00, 15.37, 14.72.

#### DTC1: 2-((piperidine-1-carbonothioyl)thio)ethyl-9-hydroxy-5*a*,5*b*,8,8,11*a*-pentamethyl-1-(prop-1-en-2-yl)icosahydro-3*aH*-cyclopenta[*a*]chrysene-3*a*-carboxylate

The product was purified by column chromatography using hexane : EtOAc (94 : 6) to afford DTC1 as a pale yellow solid, displaying a molecular ion peak at *m*/*z* 644.41 [M + H]^+^ in ESIMS, consistent with molecular formula C_38_H_61_NO_3_S_2_. ^1^H NMR (400 MHz, CDCl_3_) *δ* 4.74 (s, 1H), 4.61 (s, 1H), 4.36 (dt, *J* = 11.2, 5.7 Hz, 4H), 3.66 (t, *J* = 6.2 Hz, 2H), 3.20 (dd, *J* = 11.2, 4.9 Hz, 1H), 3.06–2.98 (m, 1H), 2.33–2.24 (m, 1H), 2.20 (td, *J* = 12.6, 3.5 Hz, 1H), 2.00–1.86 (m, 2H), 1.73 (s, 5H), 1.70 (s, 3H), 1.66–1.57 (m, 5H), 1.54–1.49 (m, 2H), 1.40 (dd, *J* = 9.6, 6.2 Hz, 8H), 1.28 (d, *J* = 9.3 Hz, 3H), 1.19–1.14 (m, 1H), 1.08–1.02 (m, 1H), 0.98 (s, 6H), 0.93 (s, 3H), 0.88 (s, 2H), 0.82 (s, 3H), 0.77 (s, 3H), 0.69 (d, *J* = 9.2 Hz, 1H); ^13^C NMR (101 MHz, CDCl_3_) *δ* 194.46, 175.92, 150.61, 109.59, 78.98, 62.07, 56.59, 55.34, 53.22, 51.34, 50.54, 49.42, 46.99, 42.42, 40.73, 38.86, 38.72, 38.34, 37.18, 37.03, 35.87, 34.33, 32.14, 30.65, 29.68, 27.99, 27.39, 26.05, 25.55, 25.41, 24.30, 20.89, 19.38, 18.28, 16.15, 16.07, 15.38, 14.72.

#### DTC2: 2-((pyrrolidine-1-carbonothioyl)thio)ethyl 9-hydroxy-5*a*,5*b*,8,8,11*a*-pentamethyl-1-(prop-1-en-2-yl)icosahydro-3*aH*-cyclopenta[*a*]chrysene-3*a*-carboxylate

The product was purified by column chromatography using hexane : EtOAc (94 : 6) to afford DTC2 as a white solid, which displayed a molecular ion peak at *m*/*z* 630.39 [M + H]^+^ in ESIMS, corresponding to molecular formula C_37_H_59_NO_3_S_2_. ^1^HNMR (400 MHz, CDCl_3_) *δ* 4.74 (s, 1H), 4.61 (s, 1H), 4.35 (dt, *J* = 11.4, 6.2 Hz, 4H), 3.91 (s, 2H), 3.66 (t, *J* = 6.2 Hz, 2H), 3.20 (dd, *J* = 11.2, 4.9 Hz, 1H), 3.10–2.98 (m, 1H), 2.44–2.15 (m, 2H), 1.95–1.89 (m, 2H), 1.66 (dt, *J* = 11.5, 9.3 Hz, 16H), 1.47–1.33 (m, 8H), 1.27 (s, 3H), 1.20–1.13 (m, 1H), 0.98 (s, 6H), 0.93 (s, 3H), 0.82 (s, 3H), 0.77 (s, 3H), 0.69 (d, *J* = 9.2 Hz, 1H); ^13^C NMR (101 MHz, CDCl_3_) *δ* 194.46, 175.94, 150.69, 109.59, 79.16, 62.07, 56.59, 55.34, 50.54, 49.42, 47.00, 42.42, 40.73, 38.86, 38.72, 38.34, 37.18, 37.03, 35.87, 34.33, 32.15, 30.65, 29.68, 27.99, 27.40, 25.55, 24.30, 20.89, 19.38, 18.28, 16.14, 16.07, 15.37, 14.72.

## Conclusions

In summary, dithiocarbamate derivatives of betulinic acid were prepared *via* a two-step reaction procedure. The method is the expeditious furnishing of products with high yields. Among all the synthesised analogs, DTC2 has emerged as the most potent antifungal derivative, displaying robust activity against *C. albicans* with an MIC and MFC of 4 μg mL^−1^. It effectively suppresses hyphal growth in a concentration-dependent manner, thereby mitigating pathogenesis. Moreover, DTC2 exhibits synergistic activity, surpassing the efficacy of individual drugs. Additionally, it demonstrates kill kinetics influenced by both time and concentration, achieving complete sterilization of *C. albicans* culture within just 8 hours at 8 times the MIC. Furthermore, DTC-2 exhibits greater efficacy against β-carbonic anhydrase with better docking scores and binding patterns than ethoxyzolamide, a well-known inhibitor of β-carbonic anhydrase.

## Data availability

The data supporting this article are included in the ESI.†

## Author contributions

Henna Amin and Hadiya Amin Kantroo have contributed equally in this work.

## Conflicts of interest

The authors declare that they have no known competing financial interest or personal relationships that could have appeared to influence the work reported in this paper.

## Supplementary Material

RA-014-D4RA05020G-s001
