# Supplementary material for: Design and synthesis of betulinic acid–dithiocarbamate conjugates as potential antifungal agents against Candida albicans
Source: RSC Adv. 2024 Dec 3;14(51):38293–301. doi: 10.1039/d4ra05020g (PMC11613976; doi:10.1039/d4ra05020g)

Supplementary Information (SI) for RSC Advances.
This journal is © The Royal Society of Chemistry 2024

# Supporting Information

**Design and Synthesis of Betulinic acid Dithiocarbamate conjugates as potential antifungal agents against *Candida albicans***

**Henna Amin,[a,c,‡] Hadiya Amin Kantroo,[b,c‡] Mohamad Mosa Mubarak,[b,c] Showkat Ahmad Bhat[a,c,] Zahoor Ahmad,[b,c*], Khursheed Ahmad Bhat,[a,c*]**

[a] Bioorganic Chemistry Division, Indian Institute of Integrative Medicine (CSIR), Srinagar, J&K, 190005 India. E-mail: kabhat@iiim.res.in
[b] Clinical Microbiology and PK-PD Division, CSIR Indian institute of Integrative Medicine, Srinagar, J&K, 190005 India. E-mail: zap@iiim.res.in
[c]Academy of Scientific and Innovative Research (AcSIR), Ghaziabad-201002, India
‡ Authors with equal contribution

**Table of Contents**


| | | |
|---|---|---|
| 1. | General information | S-3 |
| 2. | Spectral Data of Compounds | S-3 to S-8 |
| 3. | HRMS Data of Compounds | S-8 to S-11 |
| 3. | $^{1}H$ and $^{13}C$ NMR of Products | S-12 to S-23 |

## 1. General information

All the necessary chemicals and solvents were purchased from Sigma Aldrich and TCI. UV cabinet (camag) was used to visualise spots on TLC. All the products were purified using silica gel (60-120) column chromatography. $^{1}$HNMR and $^{13}$CNMR spectra were recorded in $CDCl_3$ using 400 and 125 MHZ NMR spectrometer respectively. Chemical shifts of $^{1}$H and $^{13}$C NMR were expressed in parts per million (ppm).

## 2. Spectral Data of All Compounds

**BE: 2-bromoethyl 9-hydroxy-5a,5b,8,8,11a-pentamethyl-1-(prop-1-en-2-yl)icosahydro-3aH-cyclopenta[a]chrysene-3a-carboxylate.** The product was purified by column chromatography using Hexane : EtOAc (94:6) to afford **BE** as a pale white solid, with molecular formula $C_{32}H_{51}BrO_3$ . $^{1}$H NMR (400 MHz, $CDCl_3$) δ 4.67 (s, 1H), 4.60 – 4.50 (m, 1H), 4.33 (dd, *J* = 10.9, 5.8 Hz, 2H), 3.47 (t, *J* = 5.9 Hz, 2H), 3.12 (dd, *J* = 11.2, 5.0 Hz, 1H), 3.07 – 2.85 (m, 1H), 2.25 – 2.19 (m, 1H), 2.12 (td, *J* = 12.7, 3.6 Hz, 1H), 1.89 – 1.80 (m, 2H), 1.72 (s, 1H), 1.65 (d, *J* = 2.4 Hz, 1H), 1.62 (s, 3H), 1.58 (d, *J* = 5.2 Hz, 1H), 1.53 (dd, *J* = 7.6, 3.9 Hz, 2H), 1.48 – 1.40 (m, 2H), 1.40 – 1.32 (m, 5H), 1.30 (s, 2H), 1.18 (s, 3H), 1.12 – 1.07 (m, 1H), 0.96 (d, *J* = 12.4 Hz, 1H), 0.90 (d, *J* = 2.6 Hz, 6H), 0.85 (s, 3H), 0.82 (d, *J* = 4.6 Hz, 1H), 0.75 (s, 3H), 0.68 (s, 3H), 0.61 (d, *J* = 9.0 Hz, 1H); $^{13}$C NMR (101 MHz, $CDCl_3$) δ 175.76, 150.46, 109.70, 79.02, 63.35, 56.69, 55.34, 50.55, 49.42, 46.96, 42.41, 40.74, 38.86, 38.79, 38.34, 37.19, 37.01, 34.31, 32.07, 30.59, 29.68, 29.19, 27.99, 27.38, 25.53, 20.89, 19.38, 18.29, 16.15, 16.00, 15.37, 14.72.

**DTC1: 2-((piperidine-1-carbonothioyl)thio)ethyl-9-hydroxy-5a,5b,8,8,11a-pentamethyl-1-(prop-1-en-2-yl)icosahydro-3aH-cyclopenta[a]chrysene-3a-carboxylate**: The product was purified by column chromatography using Hexane : EtOAc (94:6) to afford **DTC 1** as a pale yellow solid with molecular formula $C_{38}H_{61}NO_3S_2$**.** $^{1}$H NMR (400 MHz, $CDCl_3$) δ 4.74 (s, 1H), 4.61 (s, 1H), 4.36 (dt, *J* = 11.2, 5.7 Hz, 4H), 3.66 (t, *J* = 6.2 Hz, 2H), 3.20 (dd, *J* = 11.2, 4.9 Hz, 1H), 3.06 – 2.98 (m, 1H), 2.33 – 2.24 (m, 1H), 2.20 (td, *J* = 12.6, 3.5 Hz, 1H), 2.00 – 1.86 (m, 2H), 1.73 (s, 5H), 1.70 (s, 3H), 1.66 – 1.57 (m, 5H), 1.54 – 1.49 (m, 2H), 1.40 (dd, *J* = 9.6, 6.2 Hz, 8H), 1.28 (d, *J* = 9.3 Hz, 3H), 1.19 – 1.14 (m, 1H), 1.08 – 1.02 (m, 1H), 0.98 (s, 6H), 0.93 (s, 3H), 0.88 (s, 2H), 0.82 (s, 3H), 0.77 (s, 3H), 0.69 (d, *J* = 9.2 Hz, 1H); $^{13}$C NMR (101 MHz, $CDCl_3$) δ 194.46, 175.92, 150.61, 109.59, 78.98, 62.07, 56.59, 55.34,

53.22, 51.34, 50.54, 49.42 , 46.99, 42.42, 40.73, 38.86, 38.72, 38.34, 37.18, 37.03, 35.87, 34.33, 32.14 , 30.65 29.68, 27.99 , 27.39, 26.05, 25.55, 25.41, 24.30, 20.89, 19.38, 18.28, 16.15, 16.07, 15.38, 14.72.

**DTC2: 2-((pyrrolidine-1-carbonothioyl)thio)ethyl-9-hydroxy-5a,5b,8,8,11apentamethyl-1-(prop-1-en-2-yl)icosahydro-3aH-cyclopenta[a]chrysene-3a-carboxylate**: The product was purified by column chromatography using Hexane : EtOAc (94:6) to afford **DTC 2** as a white solid with molecular formula $C_{37}H_{59}NO_3S_2$.$^1$H NMR (400 MHz, $CDCl_3$) δ 4.74 (s, 1H), 4.61 (s, 1H), 4.35 (dt, *J* = 11.4, 6.2 Hz, 4H), 3.91 (s, 2H), 3.66 (t, *J* = 6.2 Hz, 2H), 3.20 (dd, *J* = 11.2, 4.9 Hz, 1H), 3.10 – 2.98 (m, 1H), 2.44 – 2.15 (m, 2H), 1.95 – 1.89 (m, 2H), 1.66 (dt, *J* = 11.5, 9.3 Hz, 16H), 1.47 – 1.33 (m, 8H), 1.27 (s, 3H), 1.20 – 1.13 (m, 1H), 0.98 (s, 6H), 0.93 (s, 3H), 0.82 (s, 3H), 0.77 (s, 3H), 0.69 (d, *J* = 9.2 Hz, 1H); $^{13}$C NMR (101 MHz, $CDCl_3$) δ 194.46, 175.94 , 150.69 , 109.59, 79.16, 62.07, 56.59, 55.34, 50.54, 49.42, 47.00, 42.42, 40.73, 38.86, 38.72, 38.34, 37.18, 37.03, 35.87, 34.33, 32.15, 30.65, 29.68 27.99, 27.40, 25.55, 24.30, 20.89, 19.38, 18.28, 16.14, 16.07, 15.37, 14.72.

**DTC3:** 2-**((4-cyanopiperidine-1-carbonothioyl)thio)ethyl9-hydroxy-5a,5b,8,8,11apenta-methyl-1-(prop-1-en-2-yl)icosahydro-3aH-cyclopenta[a]chrysene-3a-carboxylate**: The product was purified by column chromatography using Hexane : EtOAc (83:17) to afford **DTC 3** as a yellowish solid with molecular formula $C_{39}H_{60}N_2O_3S_2$. $^1$H NMR (400 MHz, $CDCl_3$) δ 4.73 (s, 1H), 4.60 (s, 1H), 4.39 – 4.29 (m, 2H), 3.63 (t, *J* = 6.2 Hz, 2H), 3.19 – 3.13 (m, 1H), 3.05 – 2.98 (m, 2H), 2.93 (d, *J* = 31.7 Hz, 1H), 2.27 – 2.16 (m, 3H), 2.03 – 1.96 (m, 4H), 1.92 – 1.87 (m, 2H), 1.68 (s, 3H), 1.65 – 1.49 (m, 5H), 1.38 (dd, *J* = 17.4, 6.7 Hz, 10H), 1.29 – 1.19 (m, 4H), 1.16 (d, *J* = 10.7 Hz, 1H), 1.02 (dd, *J* = 12.9, 4.1 Hz, 1H), 0.96 (s, 6H), 0.91 (s, 3H), 0.81 (s, 3H), 0.75 (s, 3H), 0.68 (d, *J* = 9.4 Hz, 1H); $^{13}$C NMR (101 MHz, $CDCl_3$) δ 196.14, 196.14, 175.86, 175.86, 150.50, 150.50, 120.34, 120.34, 109.69, 109.69, 78.93, 78.93, 61.71, 56.58, 55.31, 50.49 , 49.37, 47.00, 42.40, 40.70, 38.77 , 38.32, 37.08, 36.15 , 34.32, 32.10, 31.53, 30.59 , 29.67, 28.13, 27.35, 26.18, 25.50, 20.88, 19.34, 18.28, 16.12, 15.40, 14.72.

**DTC5: 2-((piperazine-1-carbonothioyl)thio)ethyl-9-hydroxy-5a,5b,8,8,11a-pentamethyl-1-(prop-1-en-2-yl)icosahydro-3aH-cyclopenta[a]chrysene-3a-carboxylate:** The product was purified by column chromatography using Hexane : EtOAc (40:60) to afford **DTC 5** as a light brown gelwith molecular formula $C_{37}H_{60}N_2O_3S_2$. $^1$H NMR (400 MHz, $CDCl_3$) δ 4.75 (s,

1H), 4.62 (s, 1H), 4.38 (td, *J* = 11.4, 5.4 Hz, 3H), 4.15 (s, 1H), 3.68 (t, *J* = 6.1 Hz, 2H), 3.20 (dd, *J* = 11.1, 4.8 Hz, 1H), 3.02 (t, *J* = 8.8 Hz, 1H), 2.41 – 2.13 (m, 3H), 1.93 (dd, *J* = 11.0, 7.7 Hz, 3H), 1.70 (s, 3H), 1.61 (t, *J* = 11.4 Hz, 3H), 1.55 (s, 1H), 1.40 (dd, *J* = 18.1, 8.0 Hz, 8H), 1.32 – 1.24 (m, 6H), 1.18 (d, *J* = 10.3 Hz, 2H), 0.98 (s, 6H), 0.93 (s, 3H), 0.87 (d, *J* = 6.5 Hz, 2H), 0.83 (s, 3H), 0.77 (s, 3H), 0.71 (s, 1H); $^{13}C$ NMR (101 MHz, $CDCl_3$) δ 196.90 , 175.81, 150.48, 109.71, 78.99 , 61.63 , 56.61, 55.35 , 50.53, 49.42 , 47.01 , 42.43 , 40.74, 38.87 , 38.73, 38.34 , 37.20, 37.00 , 35.96, 34.37, 32.11, 30.62, 29.71, 29.37, 27.99 , 27.40 , 25.53, 22.70, 20.91 , 19.35, 18.32, 16.17, 16.11, 15.38, 14.72, 14.13.

**DTC7: 2-((morpholine-4-carbonothioyl)thio)ethyl-9-hydroxy-5a,5b,8,8,11a-pentamethyl -1-(prop-1-en-2-yl)icosahydro-3aH-cyclopenta[a]chrysene-3a-carboxylate:** The product was purified by column chromatography using Hexane : EtOAc (88:12) to afford **DTC 7** as yellowish viscous liquid with molecular formula $C_{37}H_{59}NO_4S_2$.$^1H$ NMR (400 MHz, $CDCl_3$) δ 4.74 (s, 1H), 4.62 (s, 1H), 4.39 (d, *J* = 6.2 Hz, 3H), 3.79 (s, 4H), 3.67 (d, *J* = 6.2 Hz, 2H), 3.20 (dd, *J* = 11.2, 4.8 Hz, 1H), 3.04 – 2.89 (m, 1H), 2.28 (dd, *J* = 8.9, 2.6 Hz, 1H), 2.25 – 2.15 (m, 1H), 1.92 (dd, *J* = 7.2, 4.2 Hz, 2H), 1.70 (s, 3H), 1.67 – 1.58 (m, 4H), 1.56 – 1.51 (m, 2H), 1.46 – 1.35 (m, 9H), 1.31 (s, 4H), 1.27 (s, 1H), 1.17 (d, *J* = 10.8 Hz, 1H), 1.07 – 1.01 (m, 1H), 0.98 (s, 6H), 0.93 (s, 3H), 0.89 (d, *J* = 7.1 Hz, 1H), 0.83 (s, 3H), 0.77 (s, 3H), 0.69 (d, *J* = 9.4 Hz, 1H); $^{13}C$ NMR (101 MHz, $CDCl_3$) δ 196.44, 175.87, 150.55, 109.67, 78.98, 61.81 , 56.59, 55.32 , 50.52, 49.39, 47.00, 42.41, 40.72, 38.86, 38.71, 38.33, 37.18, 37.02 , 35.74 , 34.33, 32.12, 30.61, 29.69 , 27.98, 27.39, 25.52, 20.89, 19.36, 18.28, 16.17, 16.09, 15.38 , 14.72.

**DTC8: 2-((4-allylpiperazine-1-carbonothioyl)thio)ethyl-9-hydroxy-5a,5b,8,8,11a-penta-methyl-1-(prop-1-en-2-yl)icosahydro-3aH-cyclopenta[a]chrysene-3a-carboxylate**: The product was purified by column chromatography using Hexane : EtOAc (88:12) to afford **DTC 8** as brownish yellow viscous liquid with molecular formula $C_{40}H_{64}N_2O_3S_2$. $^1H$ NMR (400 MHz, $CDCl_3$) δ 5.92 – 5.69 (m, 1H), 5.37 – 5.08 (m, 2H), 4.66 (d, *J* = 2.0 Hz, 1H), 4.53 (d, *J* = 1.3 Hz, 1H), 4.37 – 4.24 (m, 3H), 4.05 (d, *J* = 7.1 Hz, 1H), 3.91 (s, 1H), 3.58 (t, *J* = 6.2 Hz, 2H), 3.11 (dd, *J* = 11.2, 5.0 Hz, 1H), 2.99 (d, *J* = 6.6 Hz, 2H), 2.92 (d, *J* = 6.2 Hz, 1H), 2.49 (s, 3H), 2.25 – 2.14 (m, 3H), 1.98 (d, *J* = 2.0 Hz, 1H), 1.89 – 1.79 (m, 2H), 1.61 (s, 3H), 1.52 (t, *J* = 11.4 Hz, 4H), 1.45 (dd, *J* = 6.5, 4.1 Hz, 1H), 1.36 – 1.27 (m, 8H), 1.23 – 1.15 (m, 4H), 1.09 (dd, *J* = 9.0, 5.8 Hz, 1H), 0.95 (dd, *J* = 12.8, 4.5 Hz, 1H), 0.89 (s, 6H),

0.84 (s, 3H), 0.74 (s, 3H), 0.63 (s, 3H), 0.61 (d, $J$ = 9.2 Hz, 1H); $^{13}C$ NMR (101 MHz, $CDCl_3$) δ 195.78, 175.87, 150.55, 133.78, 119.14, 109.63, 78.97, 61.92, 61.08, 56.60, 55.34, 52.23, 50.54, 49.42, 47.00, 42.42, 40.73, 38.86, 38.72, 38.34, 37.18, 37.02, 35.90, 34.33, 32.13, 30.64, 29.69, 27.99, 27.39, 25.54, 20.90, 19.37, 18.31, 16.16, 16.10, 15.38, 14.72

**DTC9: 2-((thiomorpholine-4-carbonothioyl)thio)ethyl-9-hydroxy-5a,5b,8,8,11a-pentamethyl-1-(prop-1-en-2-yl)icosahydro-3aH-cyclopenta[a]chrysene-3a-carboxylate:** The product was purified by column chromatography using Hexane : EtOAc (94:6) to afford **DTC 9** as brownish yellow viscous liquid with molecular formula $C_{37}H_{59}NO_3S_3$. $^1H$ NMR (400 MHz, $CDCl_3$) δ 4.66 (s, 1H), 4.53 (s, 1H), 4.28 (dt, $J$ = 11.3, 5.8 Hz, 3H), 4.05 (q, $J$ = 7.1 Hz, 1H), 3.58 (t, $J$ = 6.2 Hz, 2H), 3.11 (dd, $J$ = 11.2, 4.9 Hz, 1H), 2.93 (td, $J$ = 10.8, 4.5 Hz, 1H), 2.68 (s, 3H), 2.24 – 2.16 (m, 1H), 2.11 (td, $J$ = 12.7, 3.6 Hz, 1H), 1.86 – 1.78 (m, 2H), 1.61 (s, 3H), 1.59 – 1.49 (m, 4H), 1.48 – 1.41 (m, 2H), .138 – 1.28 (m, 8H), 1.25 – 1.15 (m, 5H), 1.11 – 1.05 (m, 1H), 1.00 – 0.92 (m, 1H), 0.89 (s, 6H), 0.85 (s, 3H), 0.80 (d, $J$ = 7.0 Hz, 1H), 0.74 (s, 3H), 0.68 (s, 3H), 0.61 (d, $J$ = 9.3 Hz, 1H); $^{13}C$ NMR (101 MHz, $CDCl_3$) δ 195.72, 176.08, 150.53, 109.86, 79.38, 61.78 , 60.420, 56.60, 55.34, 50.54, 49.41, 47.01, 42.43, 40.73 , 38.86, 38.73, 38.35, 37.19, 37.02, 35.96, 34.35, 32.13, 30.63, 29.70,27.99 , 27.40, 25.55 , 21.07 , 20.98, 19.36, 18.29, 16.16, 16.08, 15.38, 14.73.

**DTC10: 2-((4-methylpiperazine-1-carbonothioyl)thio)ethyl 9-hydroxy-5a,5b,8,8,11a-pentamethyl-1-(prop-1-en-2-yl)icosahydro-3aH-cyclopenta[a]chrysene-3a-carboxylate**. The product was purified by column chromatography using Hexane :EtOAc (25:75) to afford **DTC 10**as brownish gel with molecular formula $C_{38}H_{62}N_2O_3S_2$. $^1H$ NMR (400 MHz, $CDCl_3$) δ 4.66 (s, 1H), 4.53 (s, 1H), 4.28 (td, $J$ = 6.1, 4.4 Hz, 3H), 3.98 (s, 1H), 3.57 (t, $J$ = 6.2 Hz, 2H), 3.35 (s, 1H), 3.16 – 3.10 (m, 1H), 2.96 (d, $J$ = 4.7 Hz, 1H), 2.60 – 2.54 (m, 4H), 2.35 (s, 1H), 2.37 – 2.31 (m, 3H), 2.22 – 2.10 (m, 2H), 1.98 – 1.93 (m, 1H), 1.86 – 1.82 (m, 2H), 1.61 (s, 3H), 1.52 (s, 3H), 1.48 – 1.42 (m, 1H), 1.36 – 1.27 (m, 7H), 1.22 – 1.17 (m, 4H), 1.09 (d, $J$ = 3.4 Hz, 1H), 0.96 (d, $J$ = 4.4 Hz, 1H), 0.89 (s, 6H), 0.84 (s, 3H), 0.79 (s, 1H), 0.74 (s, 3H), 0.68 (s, 3H), 0.62 (s, 1H); $^{13}C$ NMR (101 MHz, $CDCl_3$) δ 196.16, 175.61, 150.54 , 109.65 , 79.03, 61.83, 56.59 , 55.32, 53.78, 50.53, 49.40, 47.00, 44.89, 42.41, 40.72, 38.84 , 38.71, 38.33, 37.17, 37.01, 35.95, 34.32, 32.12, 30.62, 29.68, 27.97, 27.33, 25.77, 25.56 20.92 , 19.35, 18.29 , 17.63 , 16.15, 16.08 , 15.37 , 14.71.

**DTC11**: **2-((4-cyclopropylpiperazine-1-carbonothioyl)thio)ethyl-9-hydroxy-5a,5b,8,8,11a-pentamethyl-1-(prop-1-en-2-yl)icosahydro-3aH-cyclopenta[a]chrysene-3a-**

**carboxylate**: The product was purified by column chromatography using Hexane:EtOAc (94:6) to afford **DTC 11** as brownish solid with molecular formula $C_{40}H_{64}N_2O_3S_2$. $^1$H NMR (400 MHz, $CDCl_3$) δ 4.66 (s, 1H), 4.53 (s, 1H), 4.35 – 4.24 (m, 4H), 3.85 (s, 1H), 3.58 (s, 2H), 3.11 (dd, *J* = 11.0, 4.9 Hz, 1H), 2.95 (dd, *J* = 14.1, 7.1 Hz, 1H), 2.69 (d, *J* = 34.1 Hz, 5H), 2.14 (ddd, *J* = 24.2, 11.8, 5.9 Hz, 3H), 1.91 – 1.77 (m, 3H), 1.61 (s, 4H), 1.52 (s, 3H), 1.32 (d, *J* = 16.7 Hz, 8H), 1.19 (s, 4H), 1.11 – 1.05 (m, 2H), 0.95 (d, *J* = 11.3 Hz, 1H), 0.89 (s, 6H), 0.85 (s, 3H), 0.74 (s, 3H), 0.68 (s, 3H), 0.60 (d, *J* = 9.1 Hz, 1H), 0.43 (dd, *J* = 7.1, 5.2 Hz, 4H); $^{13}$C NMR (101 MHz, $CDCl_3$) δ 195.63, 175.87 , 150.54, 109.63 , 78.92, 61.94, 56.58, 55.33, 52.60, 50.52, 49.41, 46.98, 42.40, 40.72, 38.85, 38.72, 38.32, 38.02, 37.17, 37.01, 35.88, 34.32, 32.12, 30.63, 29.68, 28.00, 27.39, 25.53, 20.89, 19.38, 18.29, 16.16, 16.09, 15.40, 14.72, 5.94.

**DTC12: 2-((4-(4-nitrophenyl)piperazine-1-carbonothioyl)thio)ethyl-9-hydroxy-5a,5b,8, 8,11a-pentamethyl-1-(prop-1-en-2-yl)icosahydro-3aH-cyclopenta[a]chrysene-3a-carboxylate:** The product was purified by column chromatography using Hexane: EtOAc (73:27) to afford **DTC12**as brownish solid with molecular formula $C_{43}H_{63}N_3O_5S_2$**.** $^1$H NMR (400 MHz, $CDCl_3$) δ 8.07 (d, *J* = 9.3 Hz, 2H), 6.71 (d, *J* = 9.3 Hz, 2H), 4.65 (s, 1H), 4.53 (s, 1H), 4.30 (dd, *J* = 12.4, 6.1 Hz, 3H), 4.05 (dd, *J* = 14.3, 7.1 Hz, 2H), 3.58 (dd, *J* = 11.6, 5.8 Hz, 6H), 3.11 (dd, *J* = 11.1, 4.8 Hz, 1H), 2.93 (dd, *J* = 10.5, 6.9 Hz, 1H), 2.17 – 2.11 (m, 2H), 1.99 (d, *J* = 12.0 Hz, 1H), 1.87 – 1.80 (m, 2H), 1.61 (s, 3H), 1.52 (t, *J* = 11.5 Hz, 3H), 1.40 (d, *J* = 10.9 Hz, 2H), 1.35 – 1.26 (m, 8H), 1.19 (t, *J* = 7.1 Hz, 4H), 1.09 (d, *J* = 10.6 Hz, 1H), 0.97 – 0.92 (m, 1H), 0.88 (s,3H), 0.87 (s, 3),0.84 (s, 3H), 0.73 (s, 3H), 0.66 (s, 3H), 0.60 (d, *J* = 10.3 Hz, 1H); $^{13}$C NMR (101 MHz, $CDCl_3$) δ 196.47 , 175.84, 153.65 , 150.49 , 138.96 , 126.05 , 112.21, 109.70, 78.93, 61.71 , 56.59 , 55.30, 53.49, 50.50, 49.40 , 47.02, 45.71, 42.41, 40.72, 38.83, 38.70 , 38.33, 37.17, 37.00, 35.87, 34.34, 32.11, 30.61, 29.70, 27.97, 27.33 , 25.51, 25.51, 20.89 , 19.33, 18.30, 16.16, 16.10, 15.38, 14.72.

**DTC13: 2-((4-(4-bromophenyl)piperazine-1-carbonothioyl)thio)ethyl-9-hydroxy-5a,5b, 8,8,11a-pentamethyl-1-(prop-1-en-2-yl)icosahydro-3aH-cyclopenta[a]chrysene-3a-carboxylate:** The product was purified by column chromatography using Hexane : EtOAc (85:15) to afford **DTC 13**as yellowish solidwith molecular formula $C_{43}H_{63}BrN_2O_3S_2$. $^1$H NMR (400 MHz, $CDCl_3$) δ 7.30 (d, *J* = 8.8 Hz, 2H), 6.71 (d, *J* = 8.9 Hz, 2H), 4.66 (s, 1H), 4.53 (s, 1H), 4.35 – 4.25 (m, 3H), 4.05 (dd, *J* = 14.3, 7.1 Hz, 1H), 3.60 (t, *J* = 6.1 Hz, 2H), 3.21 (t, *J* = 4.9 Hz, 5H), 3.13 – 3.07 (m, 1H), 2.98 – 2.92 (m, 1H), 2.23 – 2.07 (m, 2H), 1.98 (s, 1H), 1.88 – 1.75 (m, 2H), 1.61 (s, 3H), 1.51 (d, *J* = 11.5 Hz, 3H), 1.41 (d, *J* = 7.8 Hz, 2H),

1.33 (dd, $J$ = 12.9, 4.6 Hz, 8H), 1.19 (dd, $J$ = 8.0, 6.1 Hz, 4H), 1.11 – 1.06 (m, 1H), 0.95 (d, $J$ = 12.8 Hz, 1H), 0.89 (s, 3H), 0.88 (s, 3H), 0.84 (s, 3H), 0.73 (s, 3H), 0.67 (s, 3H), 0.60 (d, $J$ = 10.5 Hz, 1H); $^{13}C$ NMR (101 MHz, $CDCl_3$) δ 196.16, 175.86 ,150.55 , 149.25 , 132.15 , 117.82 , 112.85, 109.66 , 78.97 , 61.82 , 56.61, 55.33 , 50.53, 49.96,49.60, 48.54 , 47.02 , 42.43 , 40.74, 38.86 , 38.72, 38.35 , 37.19, 37.01 , 35.93, 34.35 , 32.13, 30.64, 29.71, 27.98 , 27.40, 25.54 , 20.91 , 19.36 , 18.30 , 16.16 , 16.11, 15.36 , 14.73 .

**3. HRMS Data of Some products**

**DTC1**

**Single Mass Analysis**
Tolerance = 100.0 PPM / DBE: min = -1.5, max = 50.0
Element prediction: Off
Number of isotope peaks used for i-FIT = 3

Monoisotopic Mass, Even Electron Ions
25 formula(e) evaluated with 1 results within limits (up to 3 closest results for each mass)
Elements Used:
C: 0-38 H: 0-100 N: 0-1 O: 0-3 S: 0-2

DTC-1 QMI DIVISION, CSIR-IIIM JAMMU 28-Aug-2024
Xevo G2-XS QTOF YFC2015 12:28:58
280824_06 4 (0.104) 1: TOF MS ES+ 7.77e+005

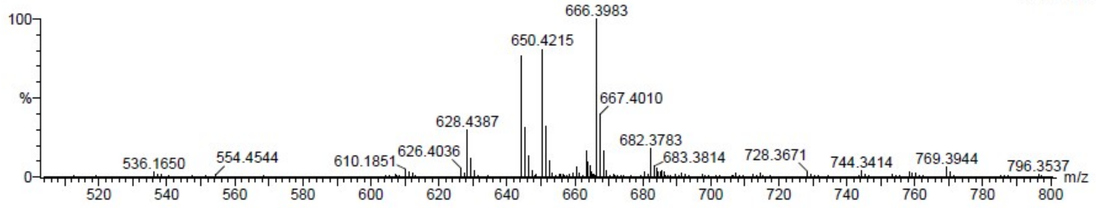


| | | | | | | | | |
|---|---|---|---|---|---|---|---|---|
| Minimum: | | | | -1.5 | | | | |
| Maximum: | | 2.0 | 100.0 | 50.0 | | | | |

| Mass | Calc. Mass | mDa | PPM | DBE | i-FIT | Norm | Conf(%) | Formula |
|---|---|---|---|---|---|---|---|---|
| 644.4167 | 644.4171 | -0.4 | -0.6 | 8.5 | 639.0 | n/a | n/a | C38 H62 N O3 S2 |

**DTC2**

**Elemental Composition Report** Page 1

**Single Mass Analysis**
Tolerance = 100.0 PPM / DBE: min = -1.5, max = 50.0
Element prediction: Off
Number of isotope peaks used for i-FIT = 3

Monoisotopic Mass, Even Electron Ions
25 formula(e) evaluated with 1 results within limits (up to 3 closest results for each mass)
Elements Used:
C: 0-37 H: 0-100 N: 0-1 O: 0-3 S: 0-2

DTC-2 QMI DIVISION, CSIR-IIIM JAMMU 28-Aug-2024
Xevo G2-XS QTOF YFC2015 12:26:16
280824_05 4 (0.104) 1: TOF MS ES+ 1.44e+006

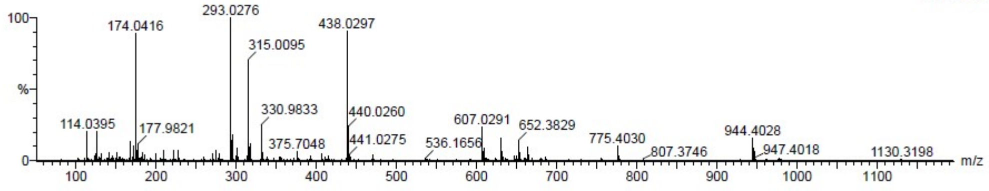


| | | | | | | | | |
|---|---|---|---|---|---|---|---|---|
| Minimum: | | | | -1.5 | | | | |
| Maximum: | | 2.0 | 100.0 | 50.0 | | | | |

| Mass | Calc. Mass | mDa | PPM | DBE | i-FIT | Norm | Conf(%) | Formula |
|---|---|---|---|---|---|---|---|---|
| 630.4012 | 630.4015 | -0.3 | -0.5 | 8.5 | 689.0 | n/a | n/a | C37 H60 N O3 S2 |

## DTC3

**Elemental Composition Report** Page 1

**Single Mass Analysis**
Tolerance = 100.0 PPM / DBE: min = -1.5, max = 50.0
Element prediction: Off
Number of isotope peaks used for i-FIT = 3

Monoisotopic Mass, Even Electron Ions
76 formula(e) evaluated with 1 results within limits (up to 3 closest results for each mass)
Elements Used:
C: 0-39 H: 0-100 N: 0-2 O: 0-3 S: 0-2 Na: 0-1

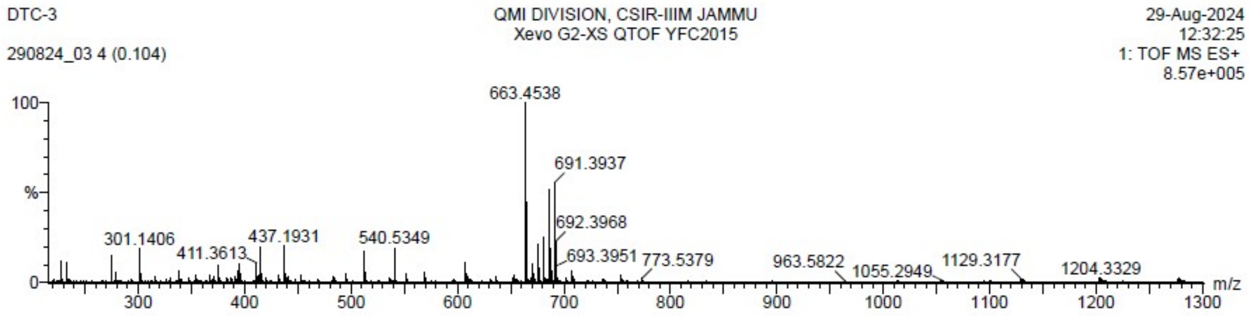


| | | | | | | | | |
|---|---|---|---|---|---|---|---|---|
| Minimum: | | | | -1.5 | | | | |
| Maximum: | | 2.0 | 100.0 | 50.0 | | | | |
| Mass | Calc. Mass | mDa | PPM | DBE | i-FIT | Norm | Conf(%) | Formula |
| 691.3937 | 691.3943 | -0.6 | -0.9 | 10.5 | 747.9 | n/a | n/a | C39 H60 N2 O3 S2 Na |

## DTC7

**Elemental Composition Report** Page 1

**Single Mass Analysis**
Tolerance = 100.0 PPM / DBE: min = -1.5, max = 50.0
Element prediction: Off
Number of isotope peaks used for i-FIT = 3

Monoisotopic Mass, Even Electron Ions
61 formula(e) evaluated with 1 results within limits (up to 3 closest results for each mass)
Elements Used:
C: 0-37 H: 0-100 N: 0-1 O: 0-4 Na: 0-1 S: 0-2

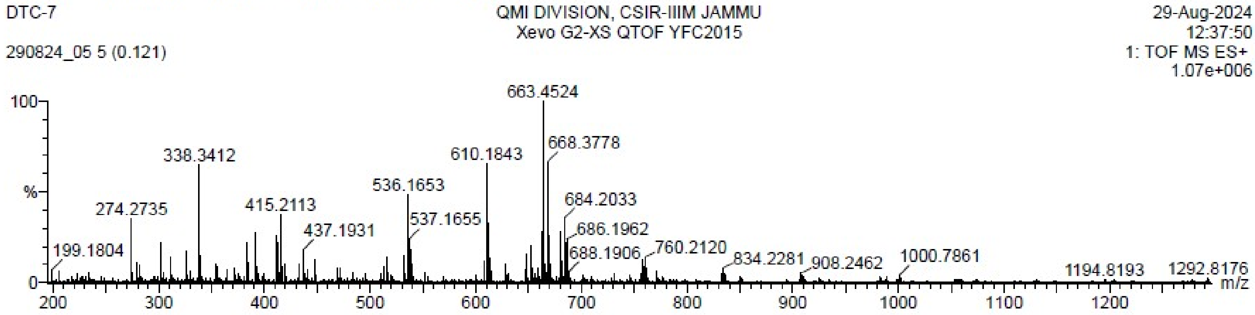


| | | | | | | | | |
|---|---|---|---|---|---|---|---|---|
| Minimum: | | | | -1.5 | | | | |
| Maximum: | | 2.0 | 100.0 | 50.0 | | | | |
| Mass | Calc. Mass | mDa | PPM | DBE | i-FIT | Norm | Conf(%) | Formula |
| 668.3778 | 668.3783 | -0.5 | -0.7 | 8.5 | 708.5 | n/a | n/a | C37 H59 N O4 Na S2 |

# DTC9

**Elemental Composition Report** Page 1

**Single Mass Analysis**
Tolerance = 100.0 PPM / DBE: min = -1.5, max = 50.0
Element prediction: Off
Number of isotope peaks used for i-FIT = 3

Monoisotopic Mass, Even Electron Ions
65 formula(e) evaluated with 1 results within limits (up to 3 closest results for each mass)
Elements Used:
C: 0-37 H: 0-100 N: 0-1 O: 0-3 Na: 0-1 S: 0-3

DTC-9 | QMI DIVISION, CSIR-IIIM JAMMU | 29-Aug-2024
Xevo G2-XS QTOF YFC2015 | 12:35:07
290824_04 4 (0.104) | 1: TOF MS ES+ | 1.72e+005

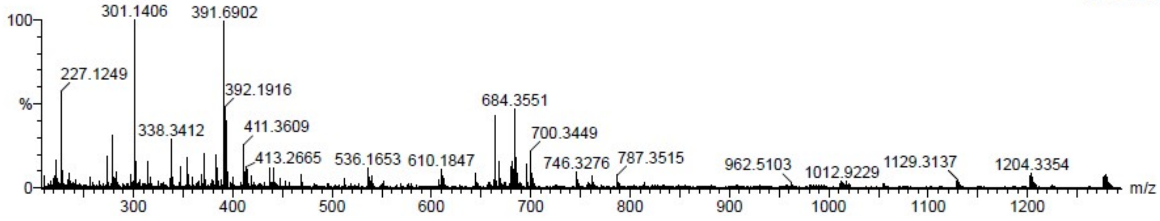


| | | | | | | | | | |
|---|---|---|---|---|---|---|---|---|---|
| Minimum: | | | | -1.5 | | | | |
| Maximum: | | 2.0 | 100.0 | 50.0 | | | | |

| Mass | Calc. Mass | mDa | PPM | DBE | i-FIT | Norm | Conf(%) | Formula |
|---|---|---|---|---|---|---|---|---|
| 684.3551 | 684.3555 | -0.4 | -0.6 | 8.5 | 592.7 | n/a | n/a | C37 H59 N O3 Na S3 |

# DTC11

**Elemental Composition Report** Page 1

**Single Mass Analysis**
Tolerance = 100.0 PPM / DBE: min = -1.5, max = 50.0
Element prediction: Off
Number of isotope peaks used for i-FIT = 3

Monoisotopic Mass, Even Electron Ions
39 formula(e) evaluated with 1 results within limits (up to 3 closest results for each mass)
Elements Used:
C: 0-40 H: 0-100 N: 0-2 O: 0-3 S: 0-2

DTC-11 | QMI DIVISION, CSIR-IIIM JAMMU | 29-Aug-2024
Xevo G2-XS QTOF YFC2015 | 12:29:52
290824_02 6 (0.138) | 1: TOF MS ES+ | 3.64e+007

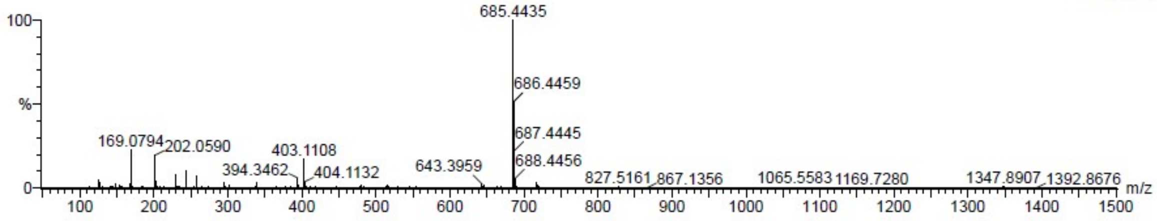


| | | | | | | | | |
|---|---|---|---|---|---|---|---|---|
| Minimum: | | | | -1.5 | | | | |
| Maximum: | | 2.0 | 100.0 | 50.0 | | | | |

| Mass | Calc. Mass | mDa | PPM | DBE | i-FIT | Norm | Conf(%) | Formula |
|---|---|---|---|---|---|---|---|---|
| 685.4435 | 685.4437 | -0.2 | -0.3 | 9.5 | 713.2 | n/a | n/a | C40 H65 N2 O3 S2 |

# DTC12

**Elemental Composition Report** **Page 1**

**Single Mass Analysis**
Tolerance = 100.0 PPM / DBE: min = -1.5, max = 50.0
Element prediction: Off
Number of isotope peaks used for i-FIT = 3

Monoisotopic Mass, Even Electron Ions
94 formula(e) evaluated with 1 results within limits (up to 3 closest results for each mass)
Elements Used:
C: 0-43 H: 0-100 N: 0-4 O: 0-5 S: 0-2

DTC-12 QMI DIVISION, CSIR-IIIM JAMMU 28-Aug-2024
Xevo G2-XS QTOF YFC2015 12:20:58
280824_03 5 (0.121) 1: TOF MS ES+
3.03e+006

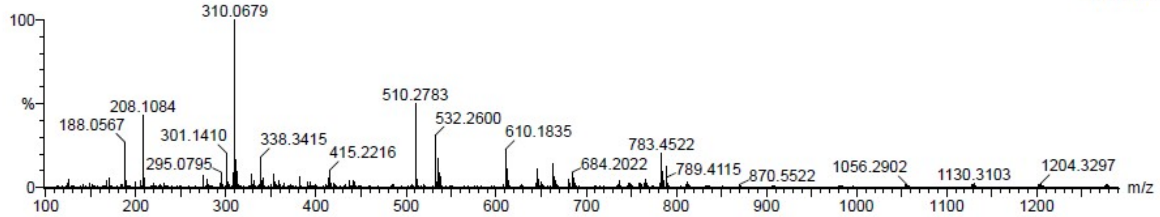


```
Minimum:                                 -1.5
Maximum:                  2.0    100.0   50.0

Mass       Calc. Mass  mDa    PPM    DBE    i-FIT   Norm   Conf(%)  Formula
783.4522   783.4553    -3.1   -4.0   12.5   601.2   n/a    n/a      C43 H67 N4 O5 S2
```

# DTC13

**Elemental Composition Report** **Page 1**

**Single Mass Analysis**
Tolerance = 100.0 PPM / DBE: min = -1.5, max = 50.0
Element prediction: Off
Number of isotope peaks used for i-FIT = 3

Monoisotopic Mass, Even Electron Ions
76 formula(e) evaluated with 1 results within limits (up to 3 closest results for each mass)
Elements Used:
C: 0-43 H: 0-100 N: 0-2 O: 0-3 S: 0-2 Br: 0-1

DTC-13 QMI DIVISION, CSIR-IIIM JAMMU 28-Aug-2024
Xevo G2-XS QTOF YFC2015 12:18:18
280824_02 4 (0.104) 1: TOF MS ES+
7.56e+006

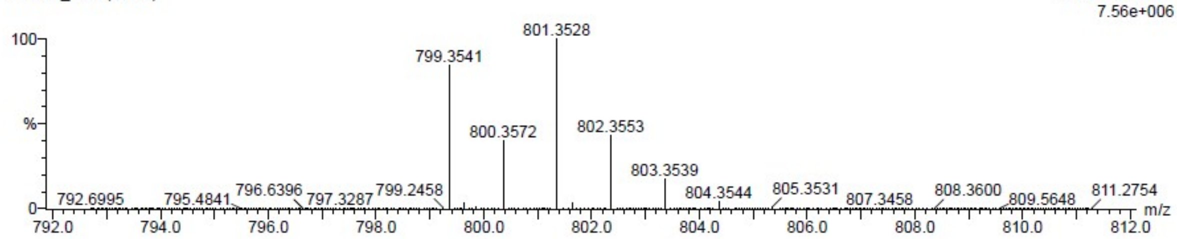


```
Minimum:                                 -1.5
Maximum:                  2.0    100.0   50.0

Mass       Calc. Mass  mDa    PPM    DBE    i-FIT   Norm   Conf(%)  Formula
799.3541   799.3542    -0.1   -0.1   12.5   592.2   n/a    n/a      C43 H64 N2 O3 S2 Br
```

## 4. $^{1}$H and $^{13}$C NMR of Products

$^{1}$H NMR and $^{13}$C NMR ($CDCl_3$) of **BE**

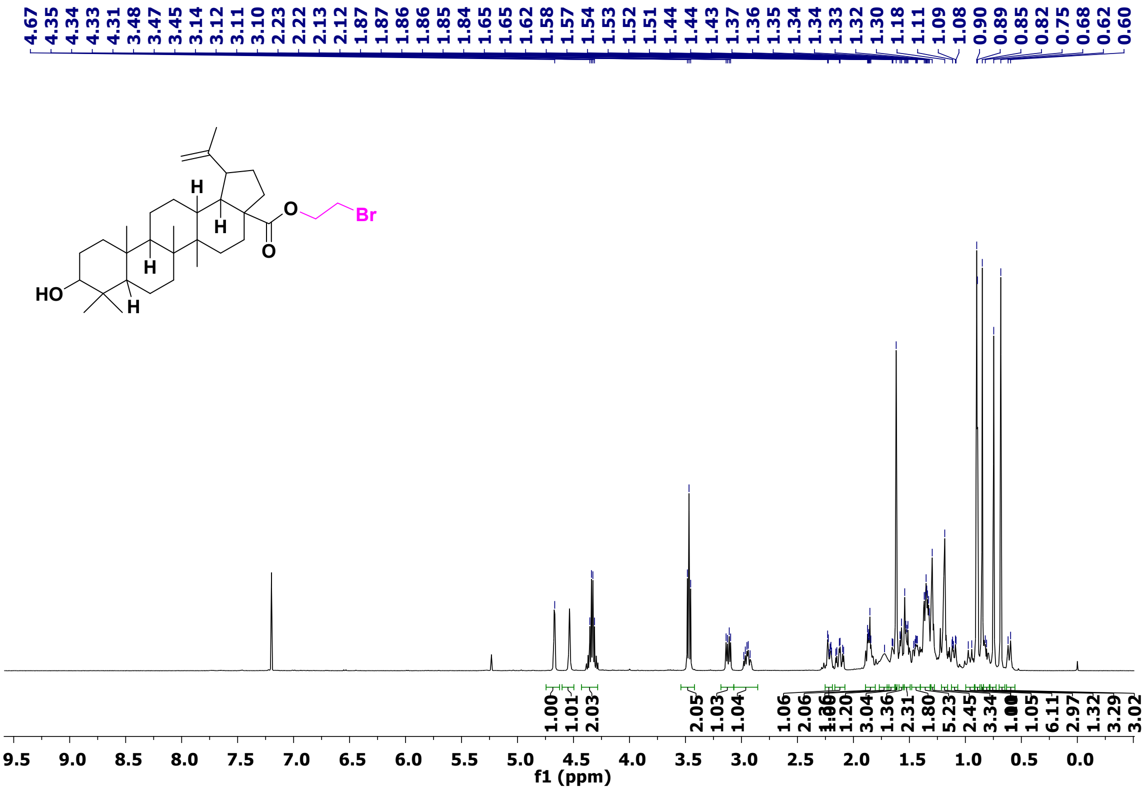


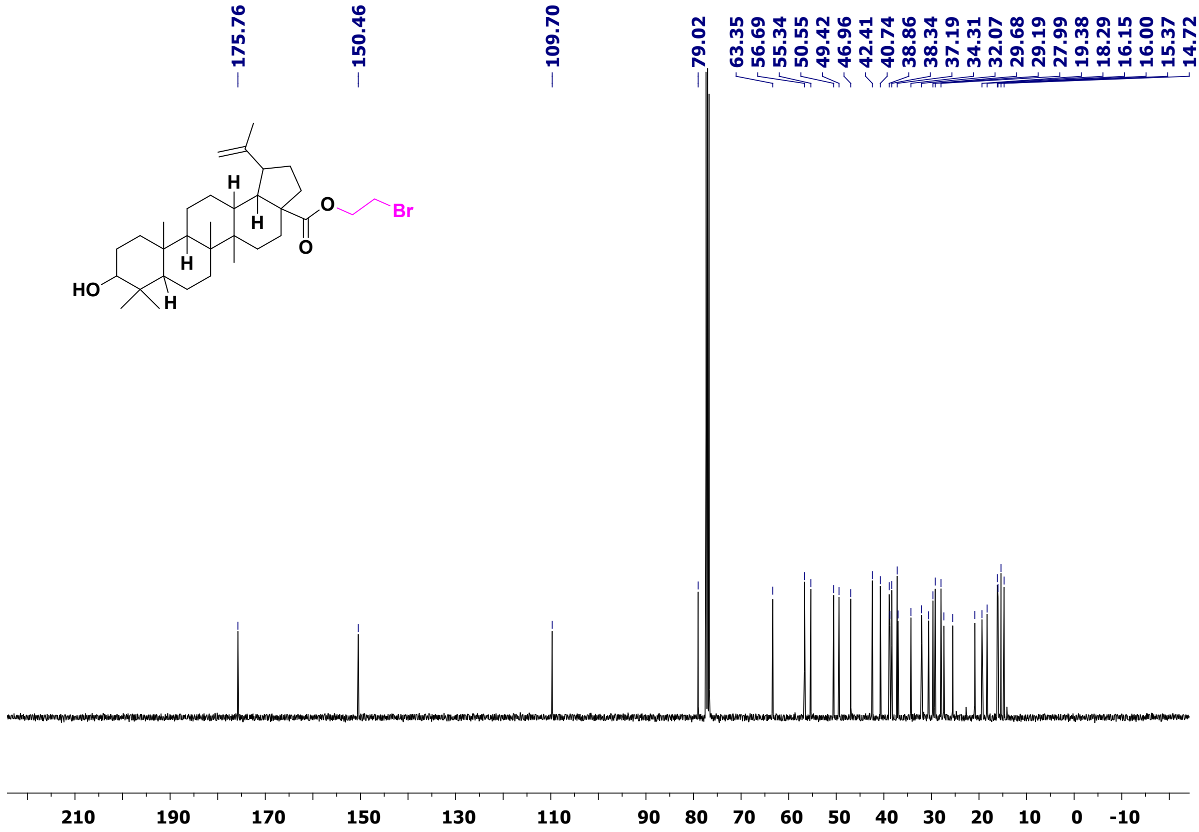

## $^{1}$H NMR and $^{13}$C NMR ($CDCl_3$) of **DTC1**

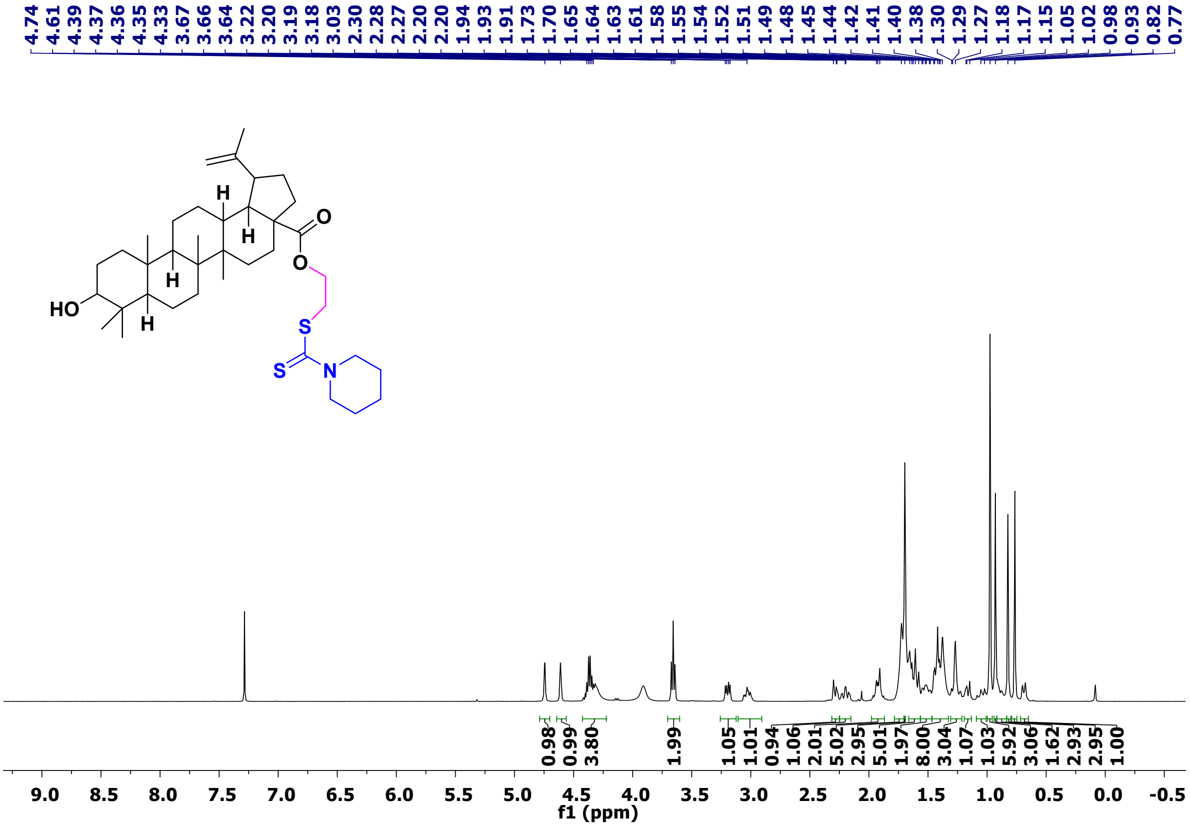


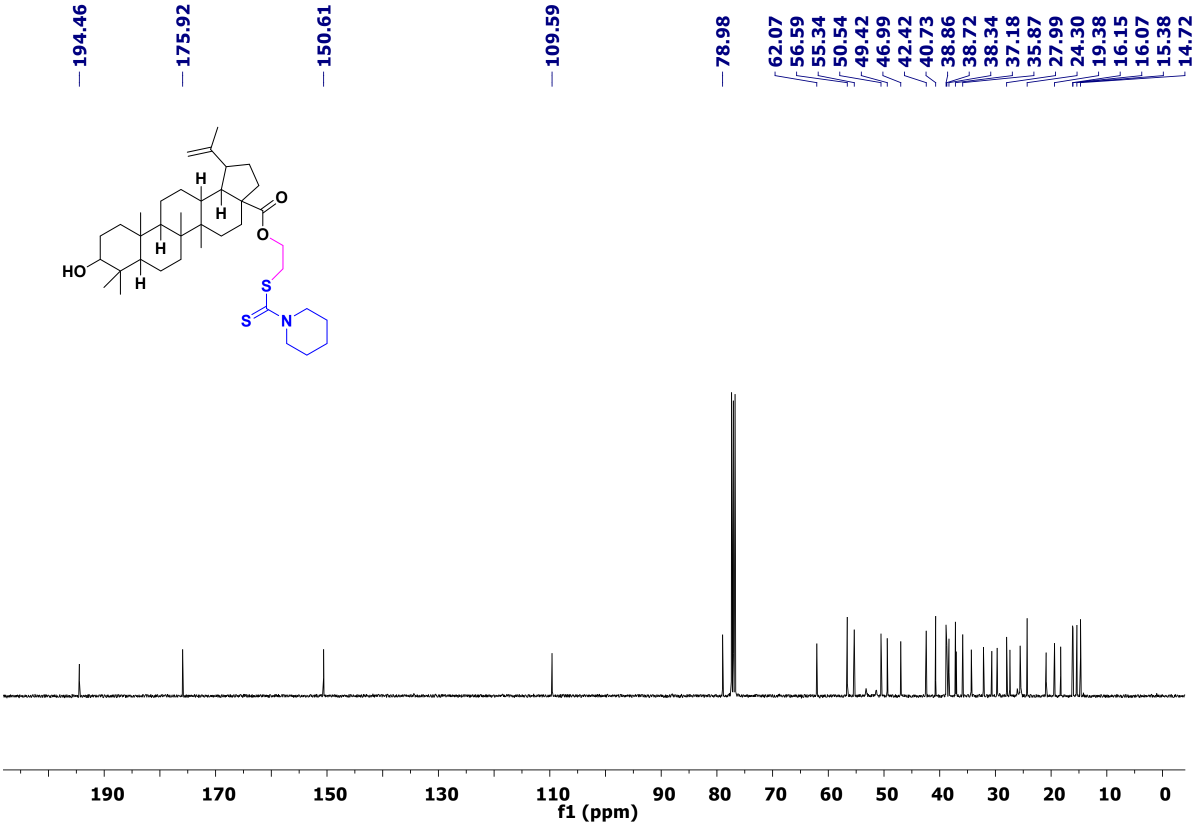

# $^{1}$H NMR and $^{13}$C NMR ($CDCl_3$) of **DTC2**

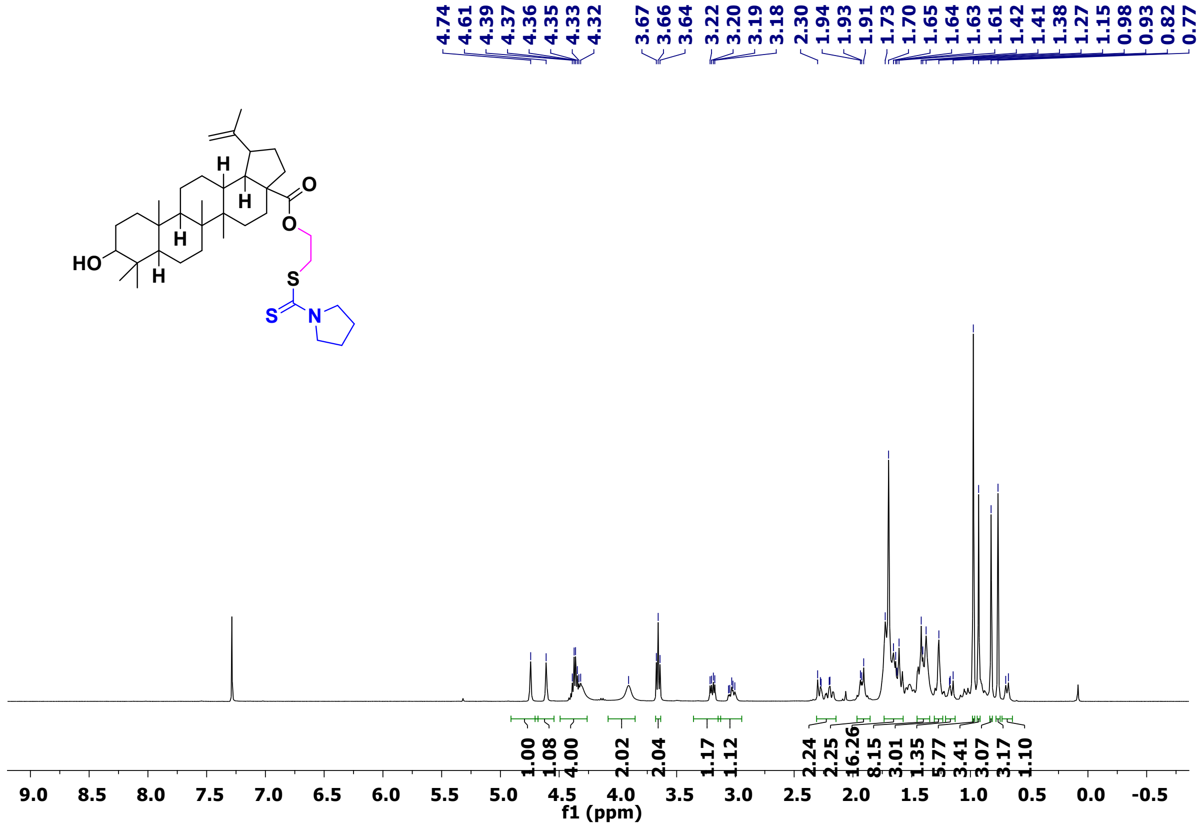


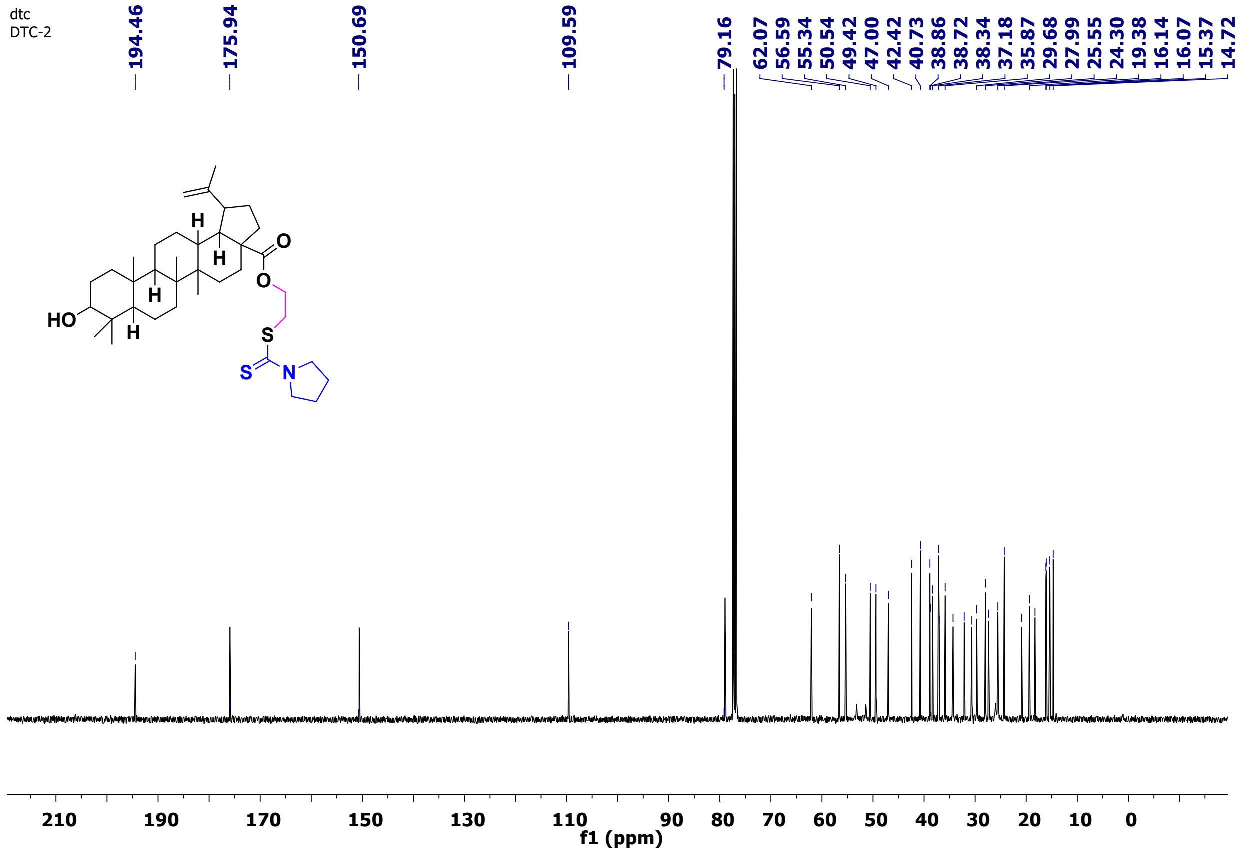

$^{1}$H NMR and $^{13}$C NMR ($CDCl_3$) of **DTC3**

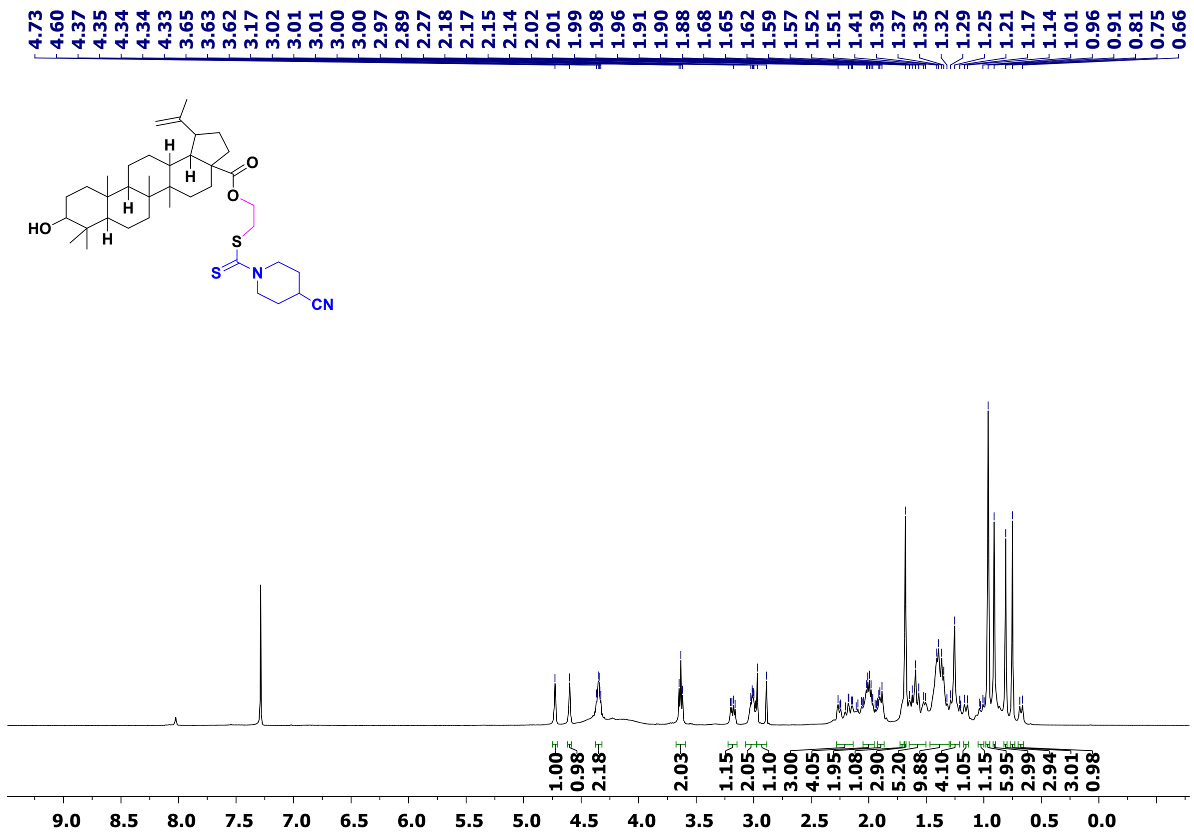


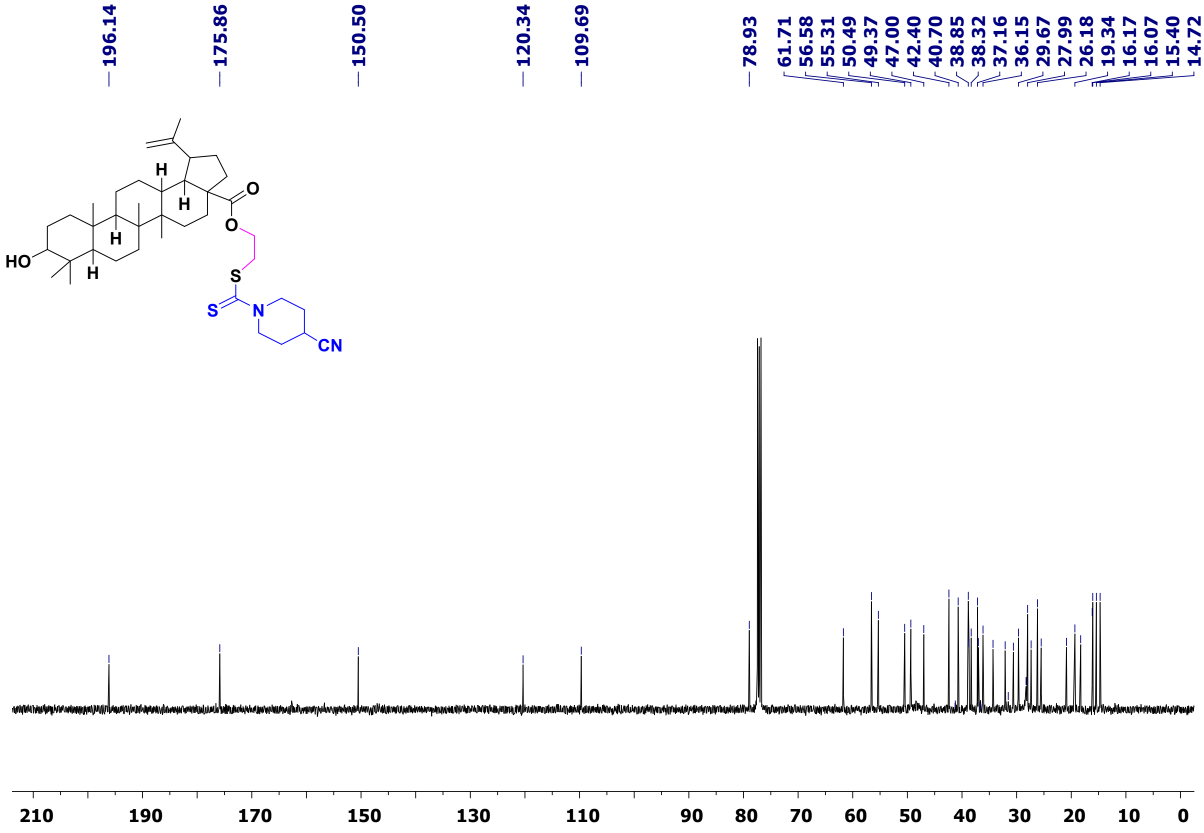

$^{1}$H NMR and $^{13}$C NMR ($CDCl_3$) of **DTC5**

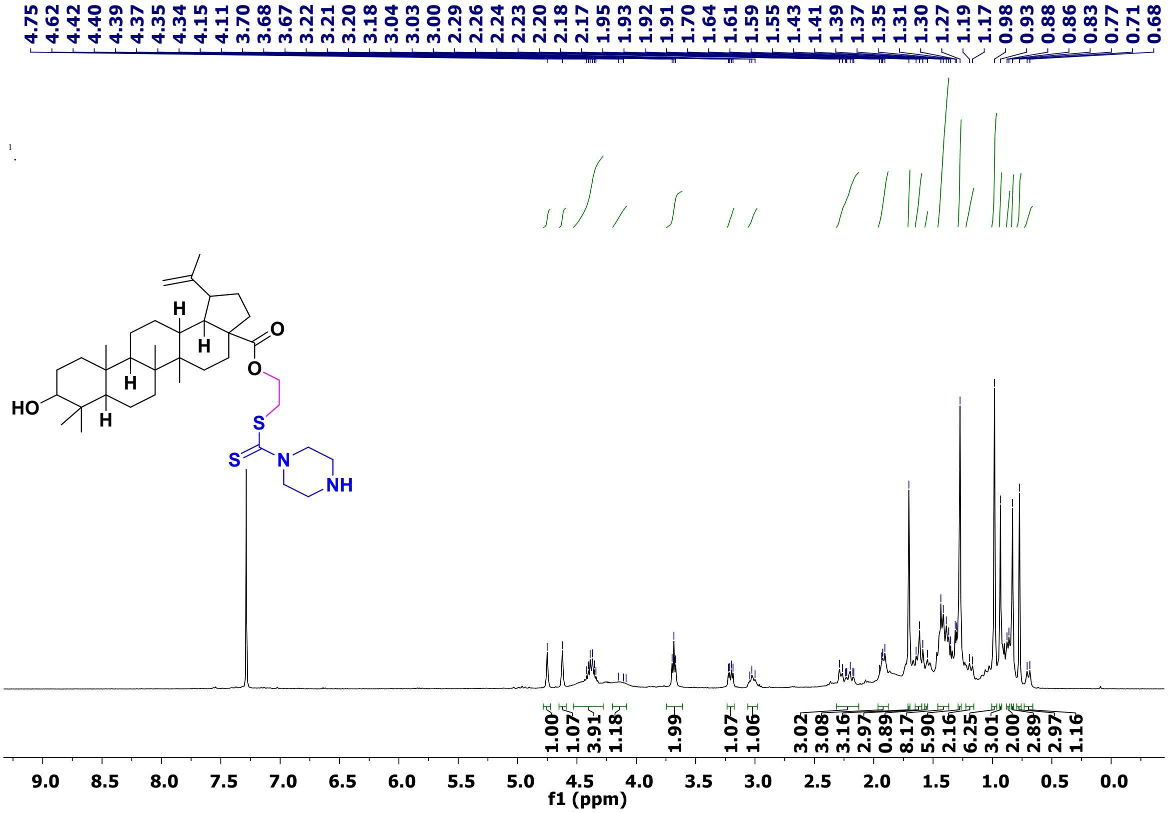


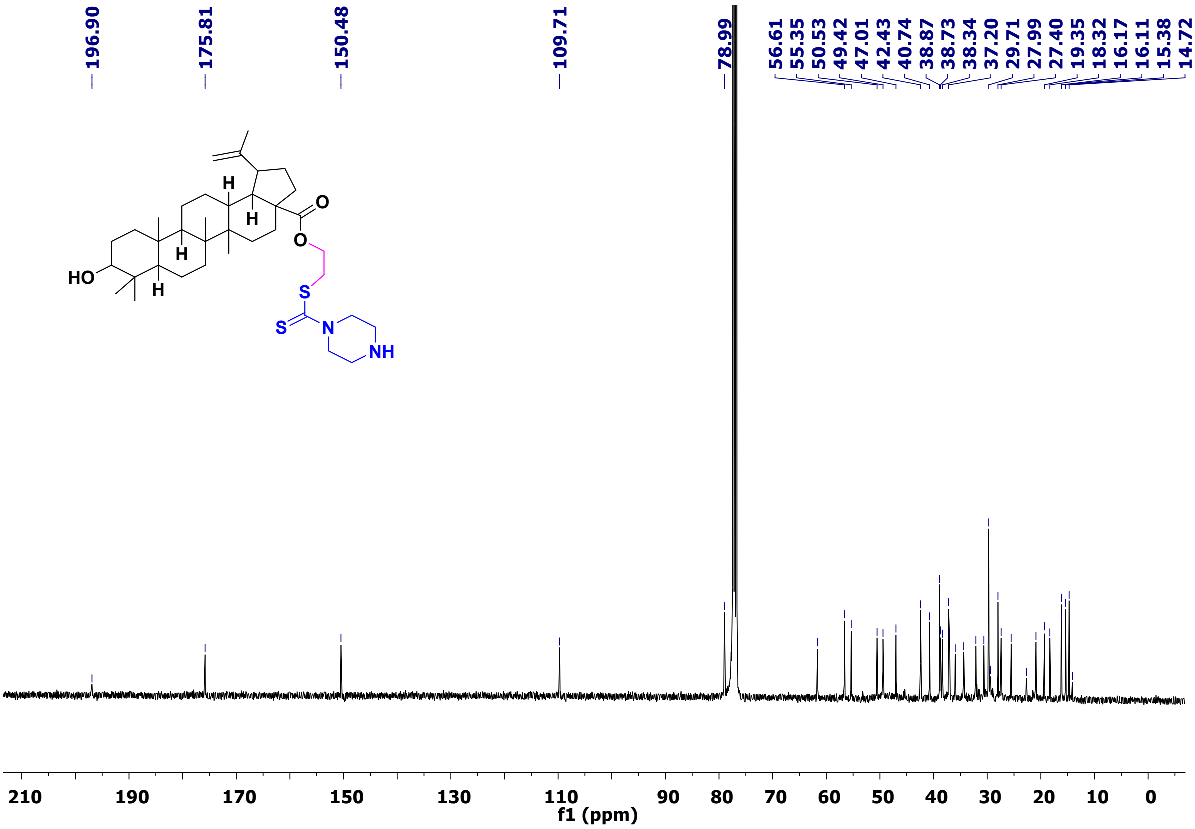

$^{1}$H NMR and $^{13}$C NMR ($CDCl_3$) of **DTC7**

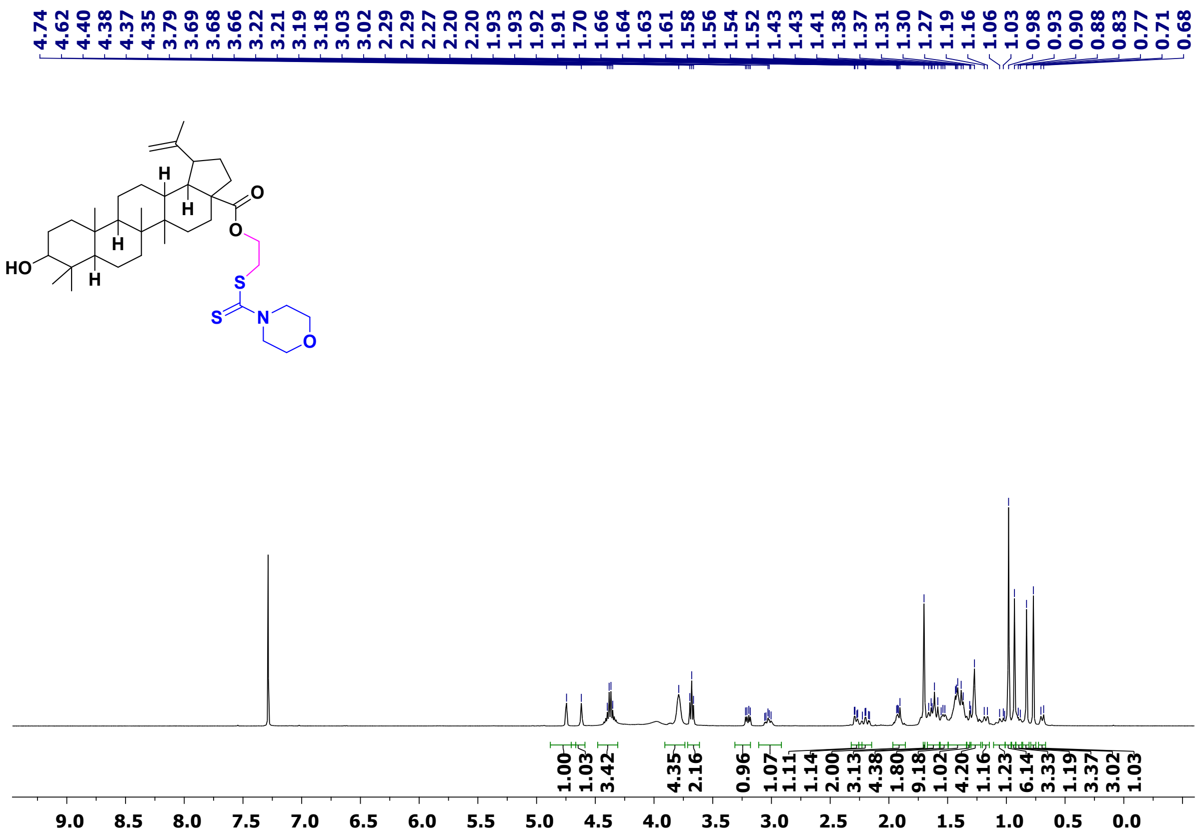

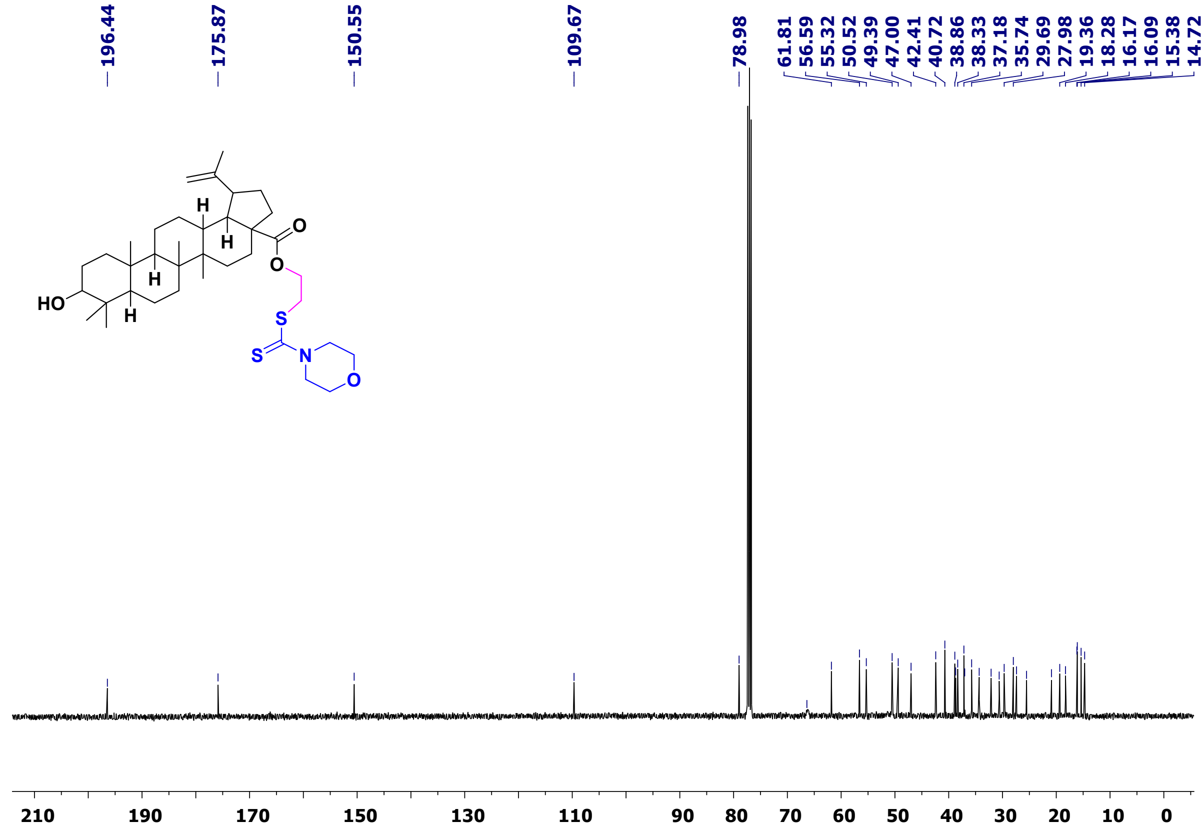

$^1H$ NMR and $^{13}C$ NMR ($CDCl_3$) of **DTC8**

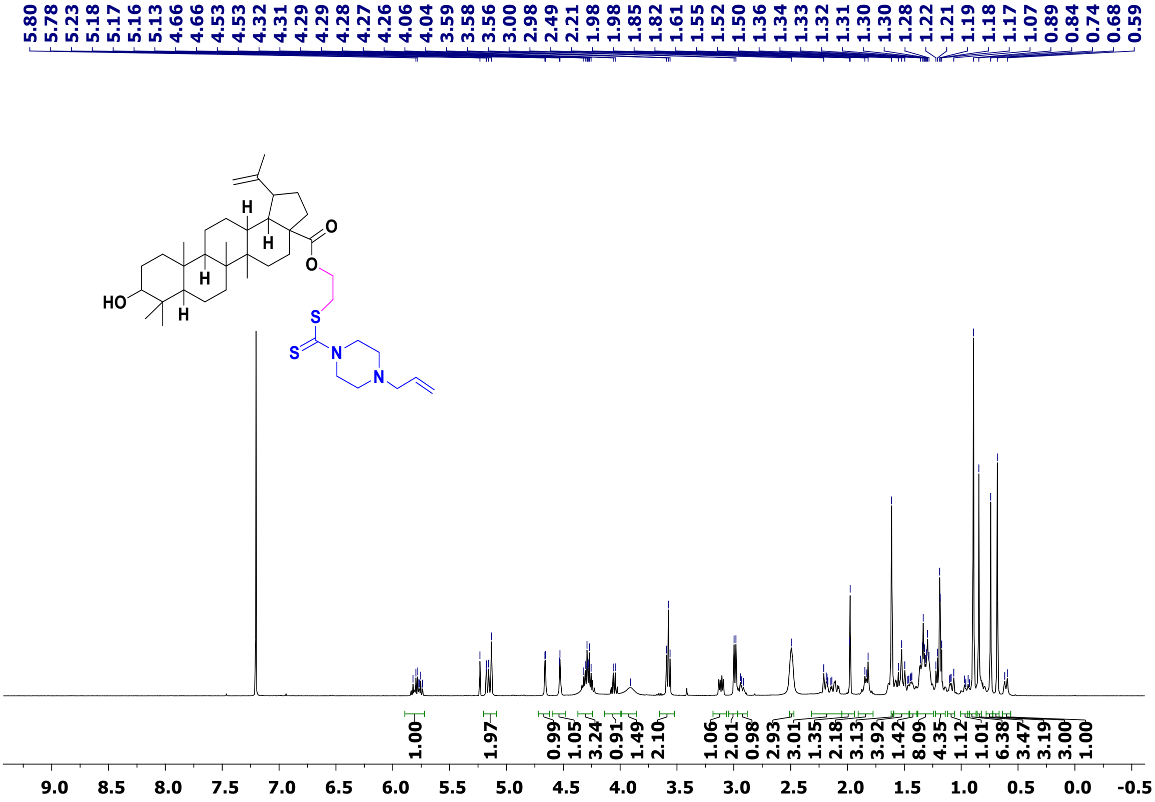


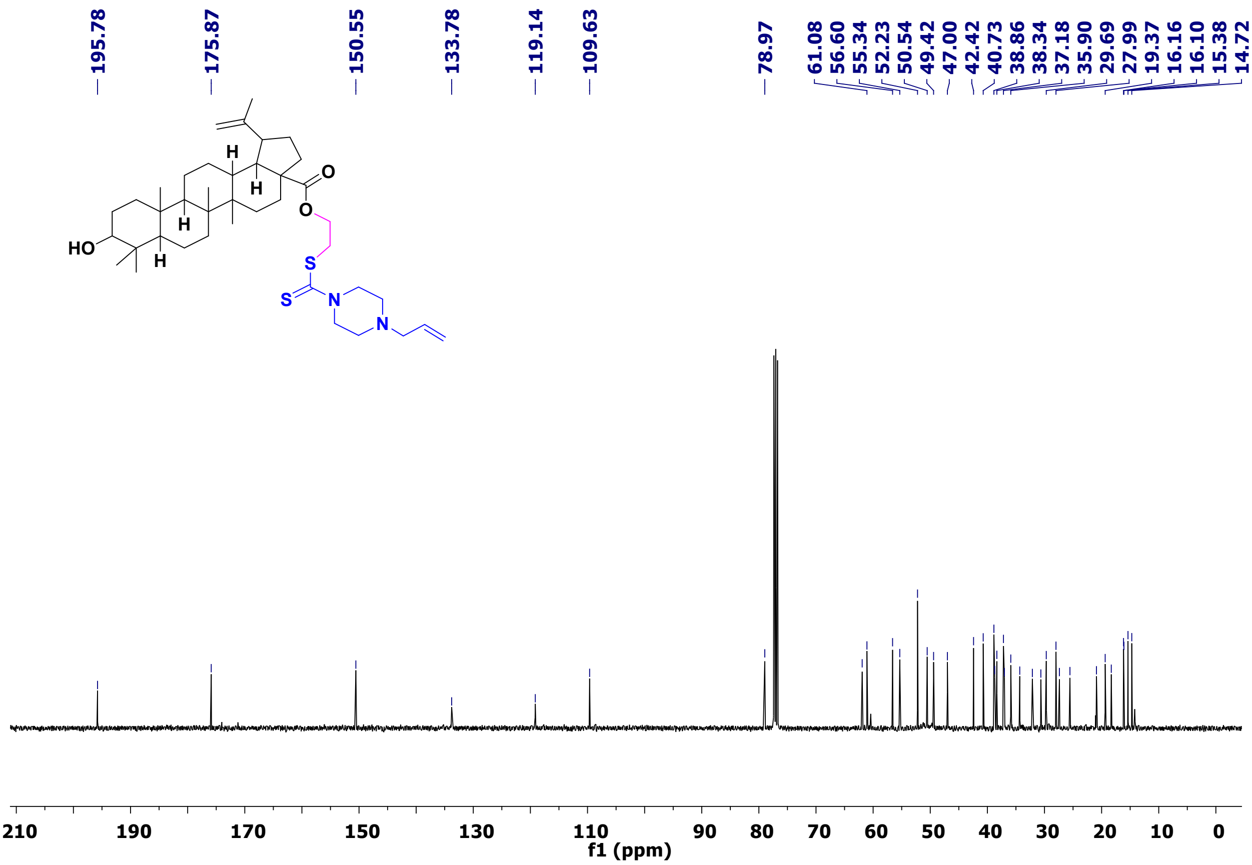

$^{1}$H NMR and $^{13}$C NMR ($CDCl_3$) of **DTC9**

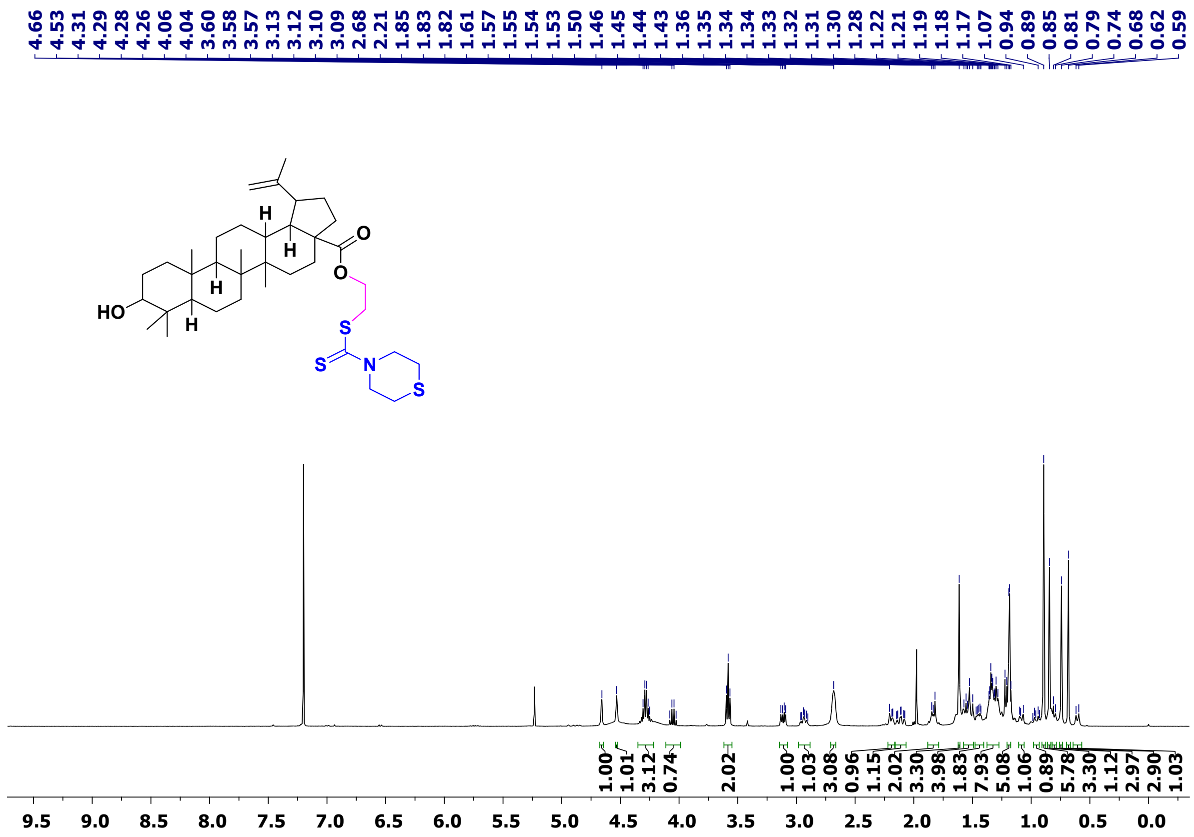


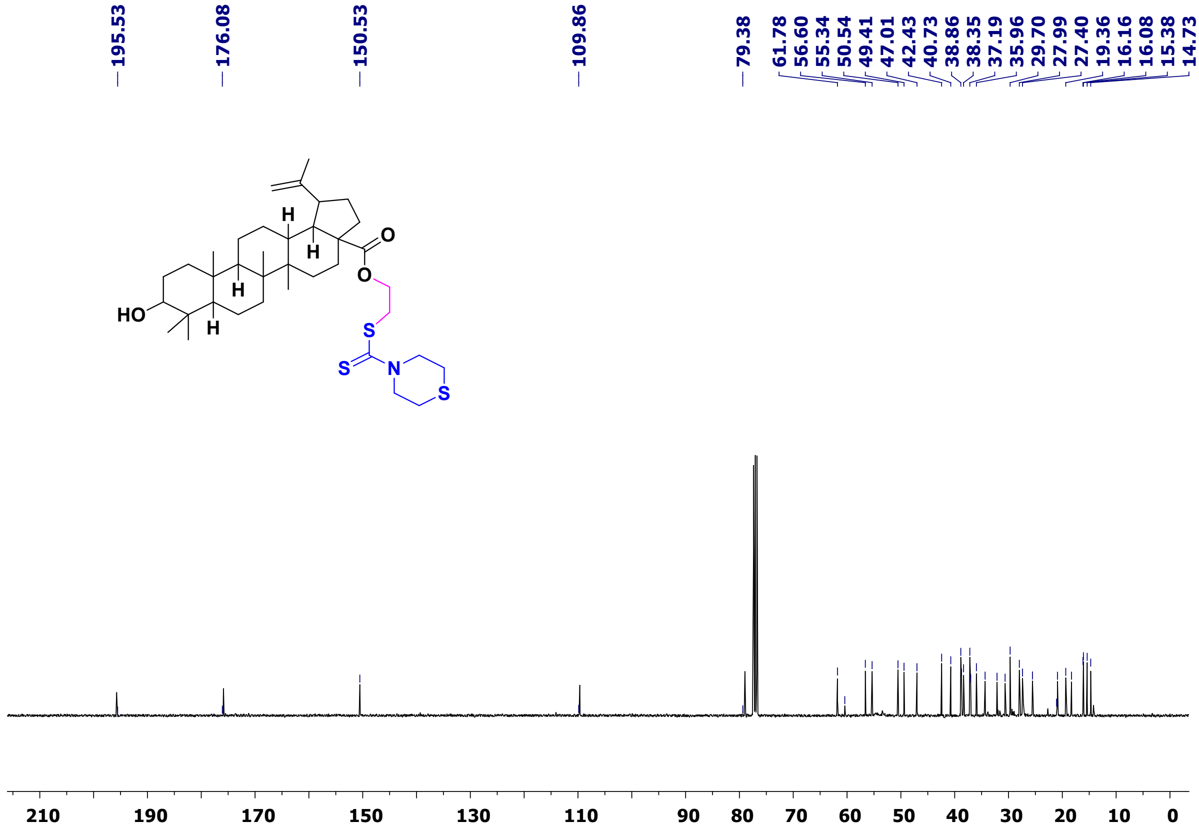

$^{1}$H NMR and $^{13}$C NMR ($CDCl_3$) of **DTC10**

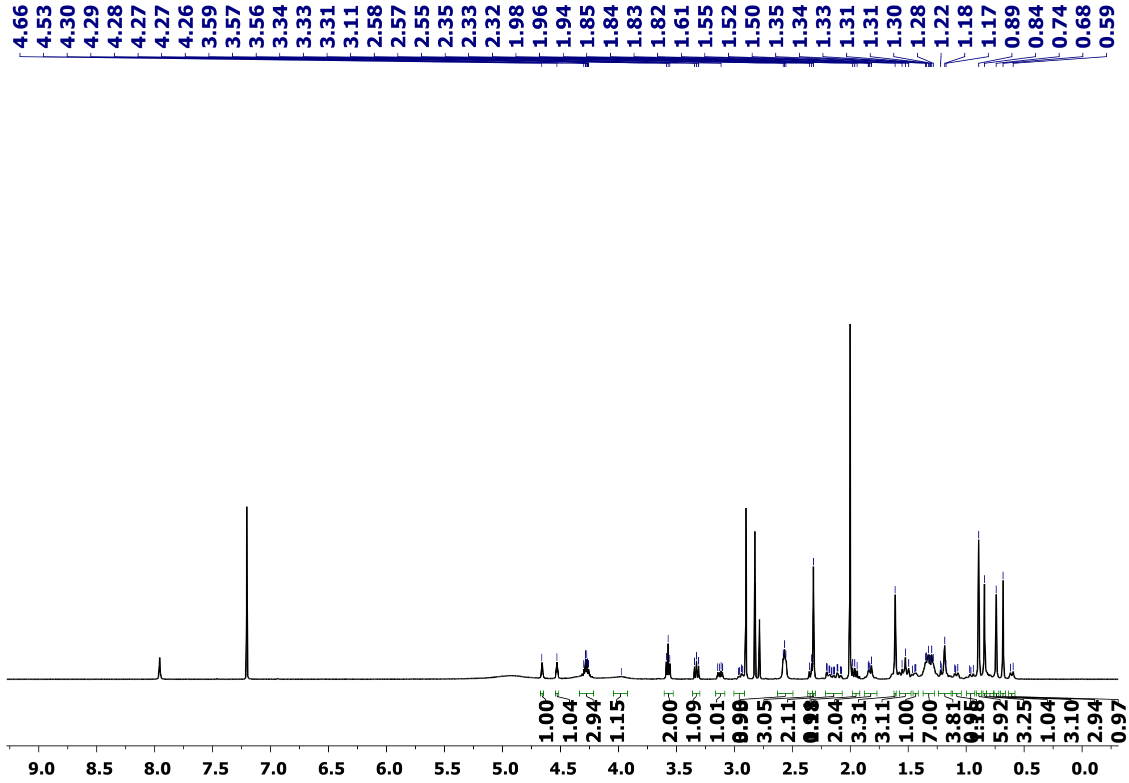


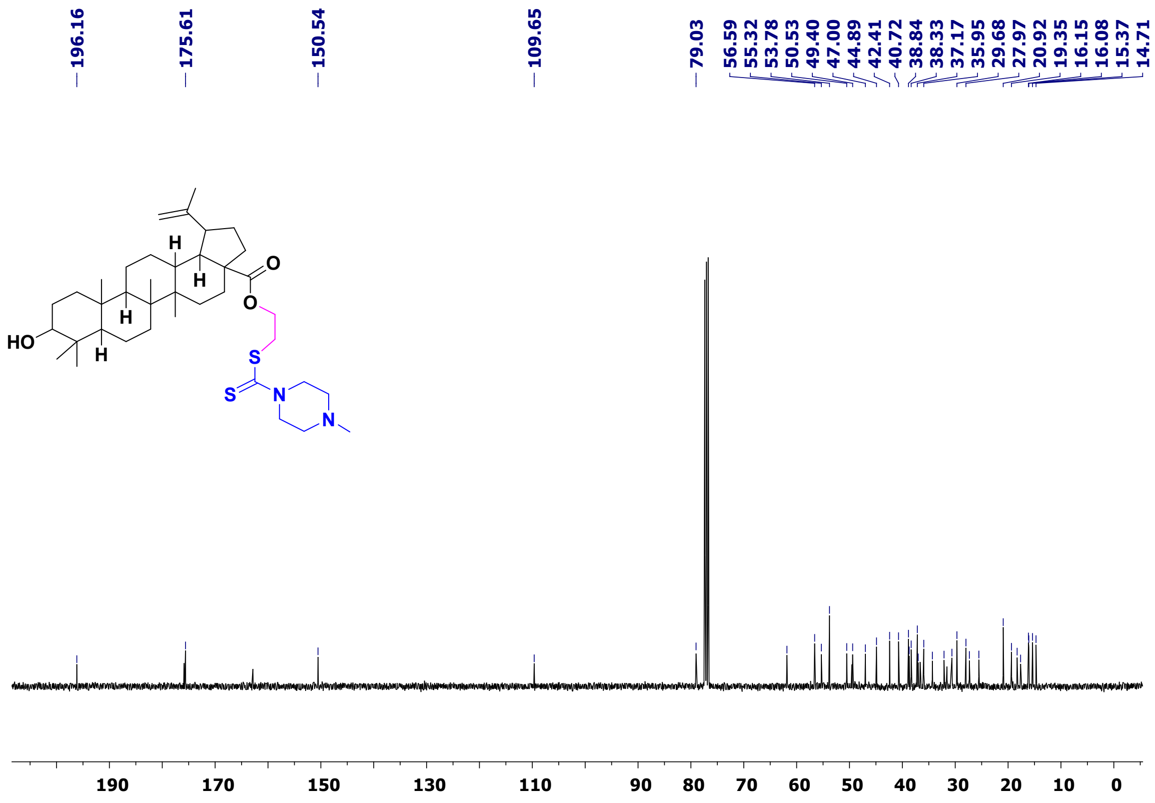

$^{1}$H NMR and $^{13}$C NMR ($CDCl_3$) of **DTC11**

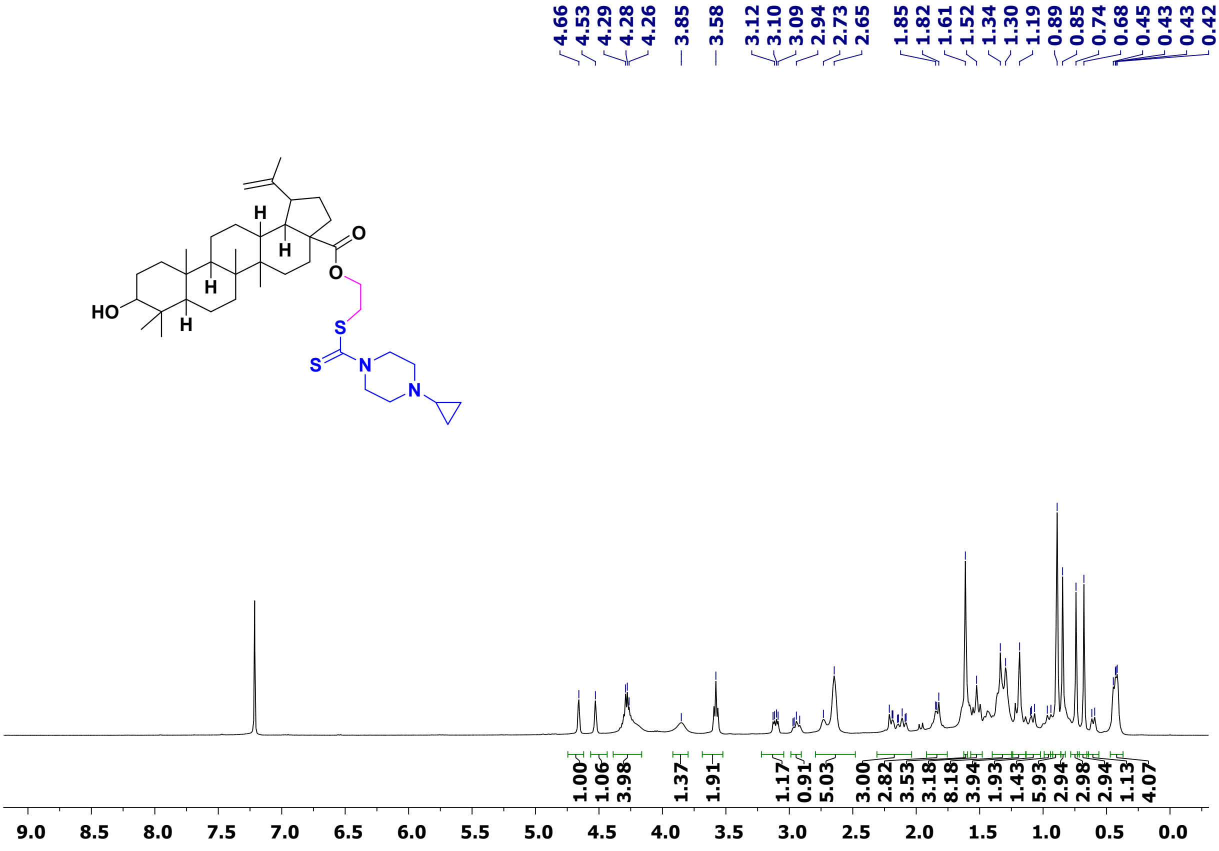


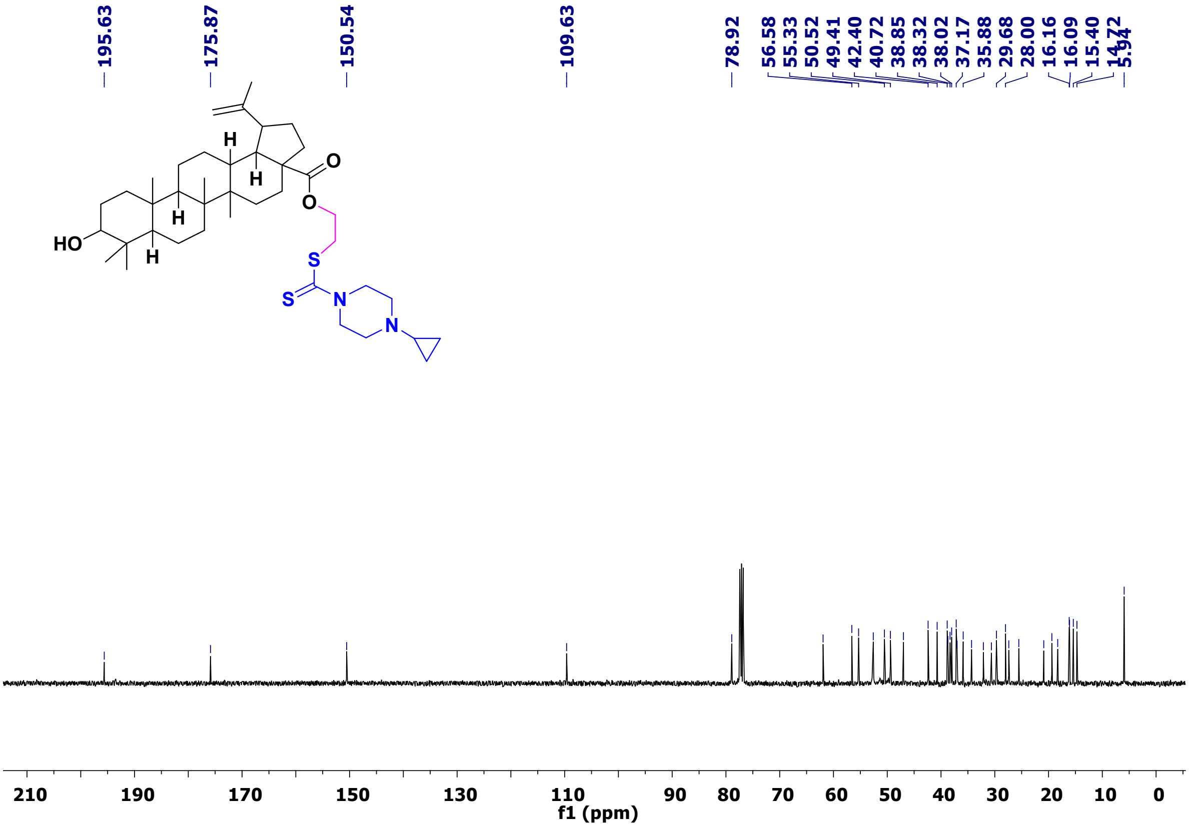

$^{1}H$ NMR and $^{13}C$ NMR ($CDCl_3$) of **DTC12**

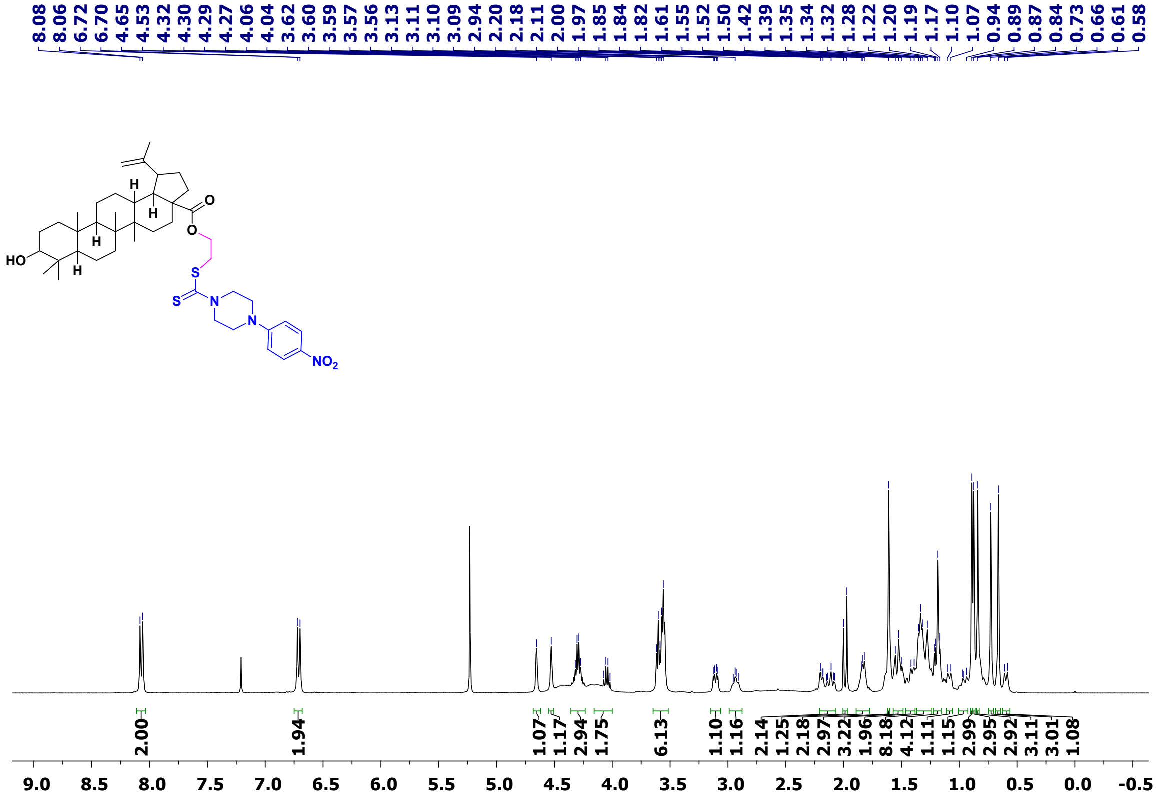


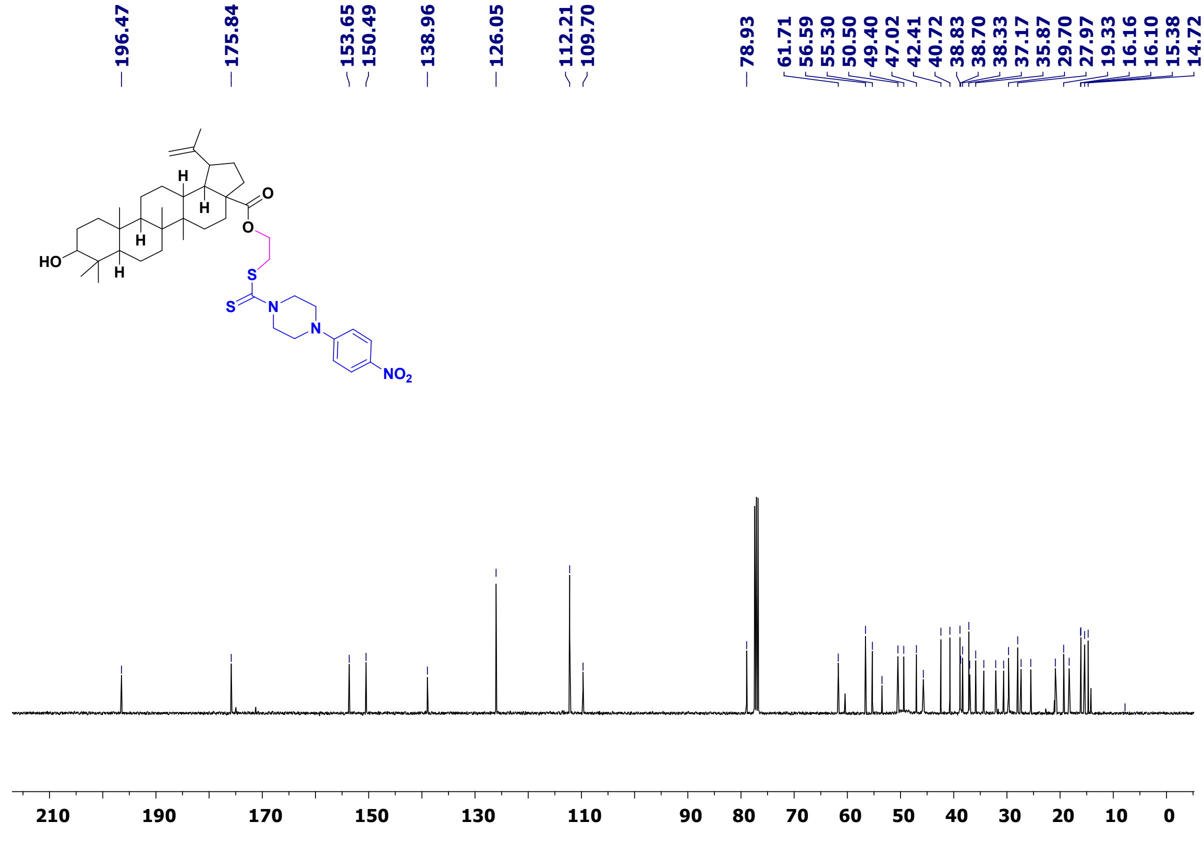

$^{1}H$ NMR and $^{13}C$ NMR ($CDCl_3$) of **DTC13**

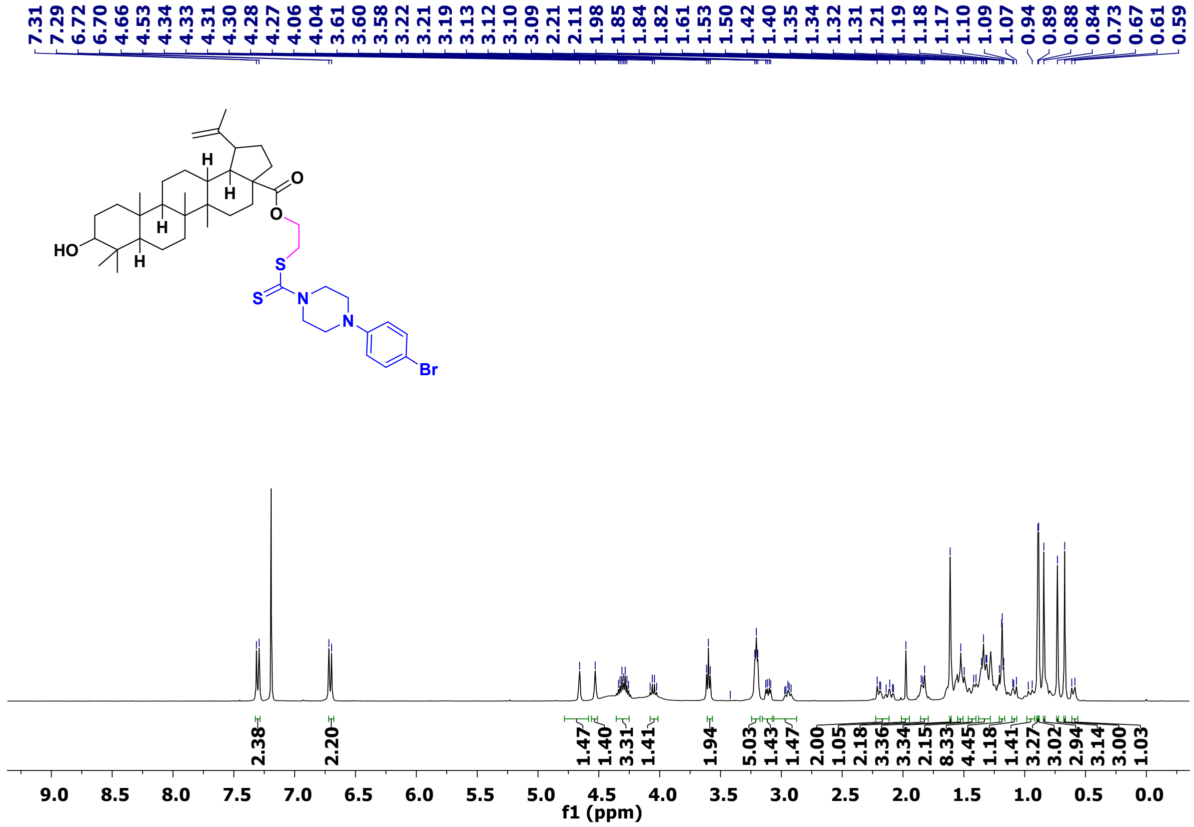


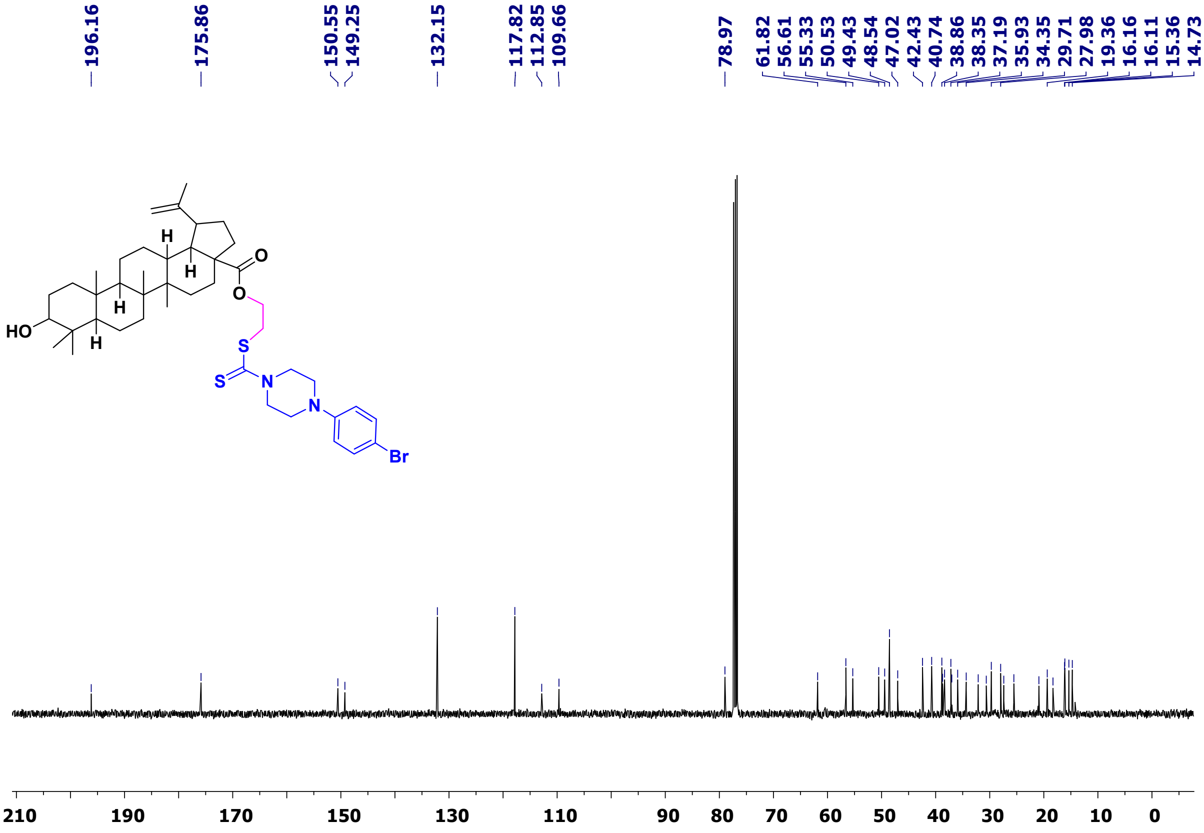

Supplement: RA-014-D4RA05020G-s001 [file RA-014-D4RA05020G-s001.pdf]
